# Deep Learning-Based Natural Language Processing in Radiology: The Impact of Report Complexity, Disease Prevalence, Dataset Size, and Algorithm Type on Model Performance

**DOI:** 10.1007/s10916-021-01761-4

**Published:** 2021-09-04

**Authors:** A. W. Olthof, P. M. A. van Ooijen, L. J. Cornelissen

**Affiliations:** 1 Department of Radiation Oncology, University of Groningen, University Medical Center Groningen, Hanzeplein 1, Groningen, The Netherlands; 2 Treant Health Care Group, Department of Radiology, Dr G.H. Amshoffweg 1, Hoogeveen, The Netherlands; 3 Hospital Group Twente (ZGT), Department of Radiology, Almelo, The Netherlands; 4 Data Science Center in Health (DASH), University of Groningen, University Medical Center Groningen, Machine Learning Lab, L.J, Zielstraweg 2, Groningen, The Netherlands; 5 COSMONiO Imaging BV, L.J, Zielstraweg 2, Groningen, The Netherlands

**Keywords:** Natural language processing, Machine learning, Informatics, Radiology

## Abstract

In radiology, natural language processing (NLP) allows the extraction of valuable information from radiology reports. It can be used for various downstream tasks such as quality improvement, epidemiological research, and monitoring guideline adherence. Class imbalance, variation in dataset size, variation in report complexity, and algorithm type all influence NLP performance but have not yet been systematically and interrelatedly evaluated. In this study, we investigate these factors on the performance of four types [a fully connected neural network (Dense), a long short-term memory recurrent neural network (LSTM), a convolutional neural network (CNN), and a Bidirectional Encoder Representations from Transformers (BERT)] of deep learning-based NLP. Two datasets consisting of radiologist-annotated reports of both trauma radiographs (n = 2469) and chest radiographs and computer tomography (CT) studies (n = 2255) were split into training sets (80%) and testing sets (20%). The training data was used as a source to train all four model types in 84 experiments (Fracture-data) and 45 experiments (Chest-data) with variation in size and prevalence. The performance was evaluated on sensitivity, specificity, positive predictive value, negative predictive value, area under the curve, and F score. After the NLP of radiology reports, all four model-architectures demonstrated high performance with metrics up to > 0.90. CNN, LSTM, and Dense were outperformed by the BERT algorithm because of its stable results despite variation in training size and prevalence. Awareness of variation in prevalence is warranted because it impacts sensitivity and specificity in opposite directions.

**Supplementary information:**

The online version contains supplementary material available at 10.1007/s10916-021-01761-4.

## Introduction

A radiology report is the primary communication method from radiologists to referring physicians [1, 2]. Radiology reports are valuable for individual patient care [3, 4] as well as the quality improvement of healthcare systems [5, 6]. At an aggregated level, anonymized radiology reports can be used to assess diagnostic yield, evaluate guideline adherence, perform epidemiological research, and be used for peer feedback and referral clinician feedback [7–11]. These applications are not widely implemented, however, because the manual classification of free-text radiology reports is cumbersome [12].

Automated processing of text is the domain of natural language processing (NLP) and has an increasing role in healthcare [13]. NLP has been applied in various applications in radiology to annotate texts or extract information [14–16]. Natural language processing has evolved from handcrafted rule-based algorithms to machine learning-based approaches and deep learning-based methods [17–24]. Deep learning is a subset of machine learning where features of the data are learned from the data by the application of multilayer neural networks [25, 26].

In machine learning, variation in size of the different classes in a dataset is called class imbalance. Together with dataset size, class imbalance potentially impacts results [27]. The impact of sample size and class imbalance is a recognized problem in machine learning in radiology but has not been fully explored [28, 29]. For NLP in medical texts, the impact of prevalence on model performance is also recognized [30]. In healthcare, the equivalent for class imbalance is called prevalence and is defined as the total number of cases of a disease at a specific point in time or during a period of time. Because the prevalence varies among different diseases and populations, class imbalance is inherent to radiological datasets. The prevalence also determines how many cases of a particular type of pathology are available for analysis in a particular population or a specific timeframe. Therefore, a prerequisite of the application of deep learning-based NLP into medical systems in clinical practice is the knowledge of the impact of prevalence and other particular characteristics of the radiology report dataset on algorithm performance. Different types of radiological examinations, different types of pathology, and different reporting styles among radiologists lead to variation in report length and complexity from a linguistic perspective. Questions that arise in the context of radiology and NLP include the following: Does variation in prevalence or variation in report complexity limit the application of NLP in radiology? What is the recommended dataset size before applying NLP in radiology? Which is the recommended algorithm to use? This study will elucidate these questions.

### Objectives

1. Build a pipeline with four different algorithm types of deep learning NLP to assess the impact of dataset size and prevalence on model performance.
2. Test this pipeline on two datasets of radiology reports with low and high complexity.
3. Formulate a best practice for deep learning NLP in radiology concerning the optimal dataset size, prevalence, and model type.

## Methods

### Study design

In this retrospective study, we developed a pipeline using Python to perform experiments to investigate the impact of data characteristics and NLP model type on binary text classification performance. The pipeline created subsets of data with variable size and prevalence and subsequently used this data to train and test four different model types. The code for the pipeline is available in the supplementary material, including a list of all used packages and their version numbers. To ensure the reproducibility of this research, we organized this paper according to the Checklist for Artificial Intelligence in Medicine (CLAIM) [31].

### Data

Two anonymized datasets of radiology reports were retrieved from the PACS of a general hospital (Treant Healthcare group, the Netherlands). The first dataset (Fracture-data) consisted of the reports (n = 2469) of all radiographs between January 2018 and September 2019 requested by general practitioners during the evening, night, and weekend shifts for patients with minor injuries to their extremities. The second dataset (Chest-data) consisted of the reports (n = 2255) of all chest radiographs (CR) and chest computed tomography (CT) studies from the first two weeks of March 2020 and the first two weeks of April 2020.

The datasets contained only the report text and annotations. No personal information about patients was included. The institutional review board confirmed that informed consent was not needed.

### Ground truth

The annotation was performed in Excel. The Fracture-data was annotated by one radiologist for the presence or absence of a fracture or other type of pathology needing referral to the emergency department. The annotations were checked for consistency by one of two other radiologists. Discrepancies (3%) were solved in consensus. The Chest-data was annotated by a single radiologist for the presence or absence of pulmonary infiltrates. For both datasets, different Dutch words (or word combinations) were attributed as positive cases. The rationale for choosing radiologists for annotation was their experience in creating radiology reports and extensive knowledge of the nuances used in radiology reporting.

Post-hoc intra-rater agreement was assessed on random sample of 15% of both datasets over one year after the initial annotation. This resulted in a Cohen’s kappa value of 0.98 for the Fracture-data and of 0.92 for the Chest-data. Appendix A1 provides examples of the annotation.

### Data partitions

Both datasets were split into separate sets of positive and negative cases. All four sets were randomized and split into training (80%) and testing (20%). The positive and negative cases of the training sets were kept separate. For both the Fracture-data and Chest-data testing sets, the positive and negative testing cases were combined.

For the Fracture-data, the training set had 1976 cases (720 positives, 1256 negatives) and the testing set had 494 cases. For the Chest-data, the training set contained 1803 cases (283 positives, 1520 negatives) and the testing set included 452 cases.

**Fig. 1:**
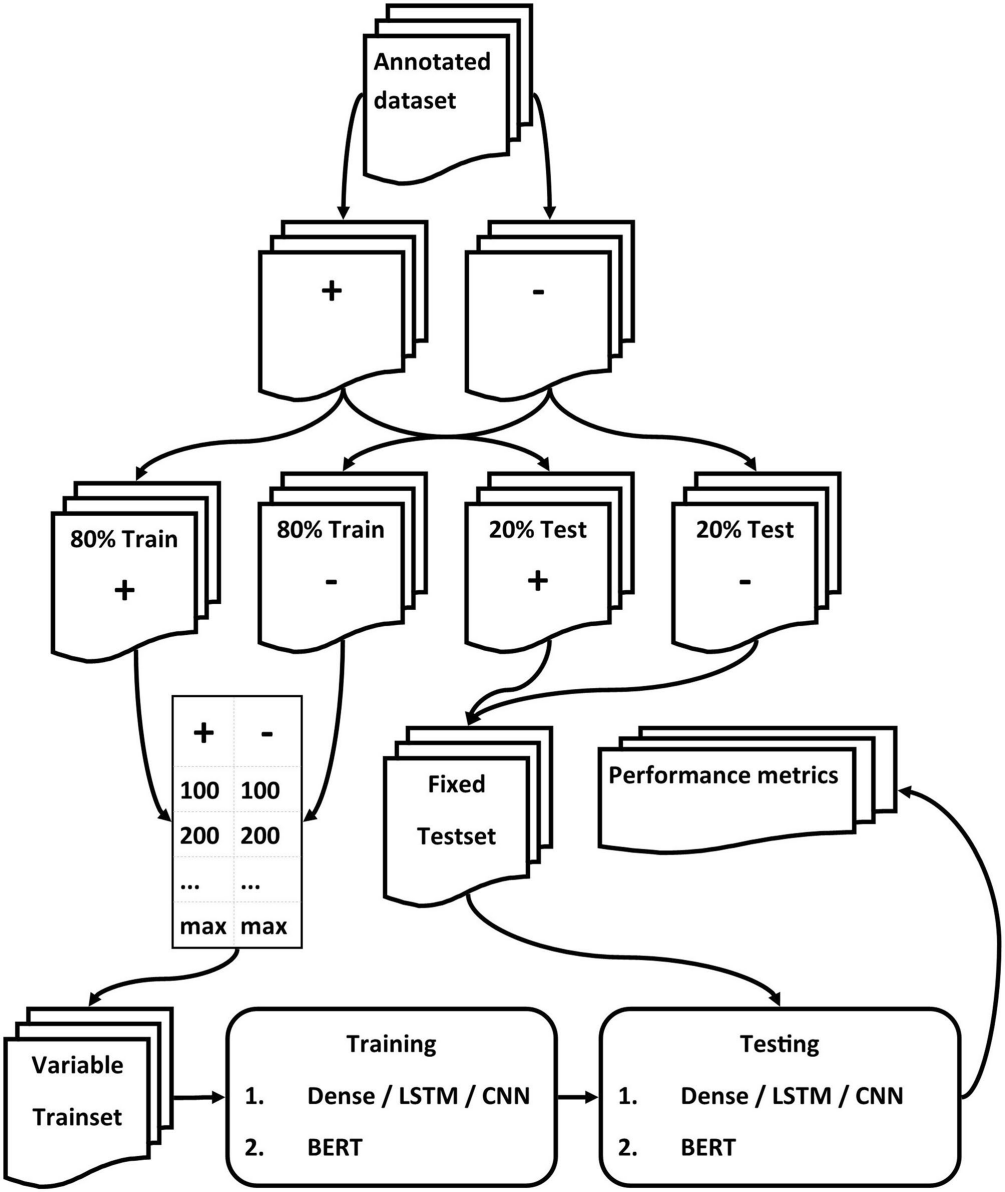
Flowchart of data processing, training, and testing. + and – refer to cases of the positive and negative classes. The input for the variable training sets are all combinations from positive and negative cases with a step size of 100. For the Fracture-data, the positive cases ranged from 100–700 and the negative cases from 100–1200. For the Chest-data, the positive cases ranged from 100–283 and the negative cases from 100–1500

The positive and negative cases were kept separate for use as sources for artificially constructed training sets with variable sizes and variable numbers of positive and negative cases. Based on the size of the datasets for both the Fracture-data and Chest-data, a list was created with all combinations of positive and negative cases using increments of 100, starting with 100 positive and 100 negative cases. For the positive cases of the Chest-data, besides 100 and 200, the largest positive number, 283, was used. These lists were used during training to create temporary training sets of a specific size. Figure 1 demonstrates the data and processing workflow.

### Models

The four models used in this study were a fully connected neural network (Dense), a bidirectional long short-term memory recurrent neural network (LSTM), a convolutional neural network (CNN), and a Bidirectional Encoder Representations from Transformers network (BERT) (Table 1).

**Table 1:** Model characteristics

Architecture	Unique characteristics and motivation	References NLP in Radiology
ANN / Dense	• Artificial neural network • No specific context awareness • Baseline model for comparative purposes	[23]
CNN	• Convolutional neural network • Well known from image classification • A sliding window (or filter or kernel) assesses the context of data. This window can be 1D (for sequential data like text), 2D (for images), of 3D (for 3D datasets or video).	[35, 39]
LSTM	• Long short-term memory • Recurrent neural network (RCN) • Designed for sequence data like text • Feedback connections transfer information from the context	[20, 24, 37, 42]
BERT	• Bidirectional Encoder Representations from Transformers • Pre-trained on massive text datasets. • Fine-tuning for specific tasks • Attention mechanism lets words focus on each other	[21, 22, 37, 38]

The Dense, LSTM, and CNN models were created using the Keras framework on top of TensorFlow 2.1.

The first layer for these three models was an embedding layer. For the Dense network, this was followed by a layer that flattened the input matrix and four fully connected layers. The LSTM network consisted of two bidirectional LSTM layers followed by two fully connected layers. The CNN network consisted of a convolutional layer, an average pooling layer, a convolutional layer, a global average pooling layer, and two fully connected layers. An overview of the three networks is provided in Appendix A2.

The number of layers and the number of epochs was empirically determined on a single training set with the original distribution of positive and negative cases for both the Fracture-data and Chest-data.

The BERT network was built using the simple transformers library in Python. BERT makes use of transfer learning, where models are pre-trained on a large text corpus in a particular language that can be fine-tuned for specific tasks [32]. In this project, the pre-trained Dutch language model 'wietsedv/bert-base-dutch-cased' was used from the Huggingface repository [33, 34]. Table 2 presents the model hyperparameters and the hardware used.

**Table 2:** Model hyperparameters for the ANN/Dense, CNN and LSTM models implemented with sequential layers in Keras (a) and for the BERT model implemented with simple transformers (b), and the hardware used for training (c)

**a. ANN/Dense, CNN, LSTM**	
**Parameter**	**Value or comment**
Vocabulary size	2500
Embedding dimension	32
Input length	150 (Fracture-data), 250 (Chest-data)
Batch size	Default (32)
Loss function	Binary cross entropy
Weigths for the loss	None
Weight regularization	None
Dropout	No dropout layers were applied in the final model
Optimizer	Adam, default parameters - learning rate 0.01 - no learning rate schedule
Epochs	12
**b. BERT**	
**Parameter**	**Value or comment**
Learning_rate	4e-5
Model_type	bert
Model_name	wietsedv/bert-base-dutch-cased
Num_train_epochs	4
Sliding_window	False
Train_batch_size	8
Use_cuda	False
Use_early_stopping	False
Weight	None
All other parameters	(also) default
**c. Hardware used for training**	
Processor	Intel Core i7, 2.20 GHz
RAM	16 GB
GPU	NVIDIA Geforce GTX 1050, 4 GB

### Training

The training was performed in four steps using the following combinations of data and models:

1. Fracture-data, Dense/LSTM/CNN
2. Fracture-data, BERT
3. Chest-data, Dense/LSTM/CNN
4. Chest-data, BERT

For each step, the models were trained multiple times using the above-mentioned temporary training sets with different sizes and prevalence. The number of epochs was empirically determined by a test run, resulting in 12 epochs for the Dense/LSTM/CNN models and four epochs for the BERT model. For the Fracture-data, 84 experiments for each model were performed; for the Chest-data, 45 experiments were performed for each model.

### Evaluation

The class imbalance for the training sets was indicated by the imbalance ratio, defined as the size ratio of the majority and minority classes.

Model performance was evaluated by assessing sensitivity, specificity, negative predictive value (npv), positive predictive value (ppv), area under the curve (auc), and F score on the fixed holdout test set from the Fracture-data (prevalence 0.36) and Chest-data (prevalence 0.16). No testing on an external dataset was performed.

The performance metrics were compared for each model using the t-test. The value *p* < 0.05 was considered to be statistically significant. Pearson correlation coefficients were calculated for training size, training prevalence, and all performance metrics for the Fracture-data and Chest-data sets.

## Results

### Data

**Fig. 2:**
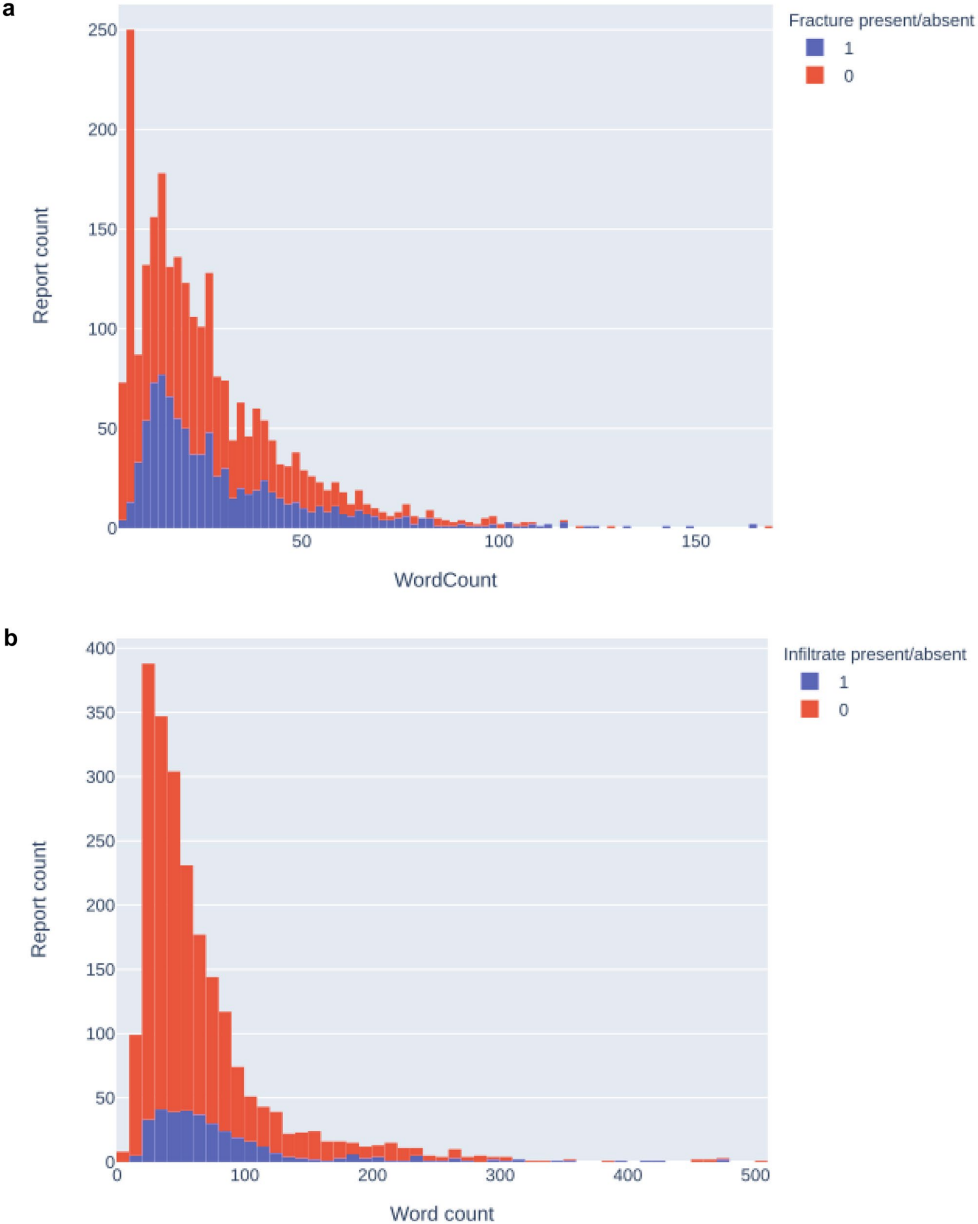
Stacked histogram demonstrating report size and binary distribution of (**a**) Fracture-data (1 = fracture present, 0 = fracture absent) and (**b**) Chest-data (1 = infiltrate present, 0 = infiltrate absent)

Figure 2 demonstrates the distribution of report word count for both Fracture-data and Chest-data. The Chest-data is more complex because of the larger variation in report size and lower prevalence of positive cases. The prevalence varies for the training sets of the Fracture-data from 0.08–0.88; for the Chest-data, from 0.06–0.74. The imbalance ratios of the Fracture-data and the Chest-data training sets range from 7.3–11.5 and from 2.9–15.7, respectively.

### Model performance

**Fig. 3:**
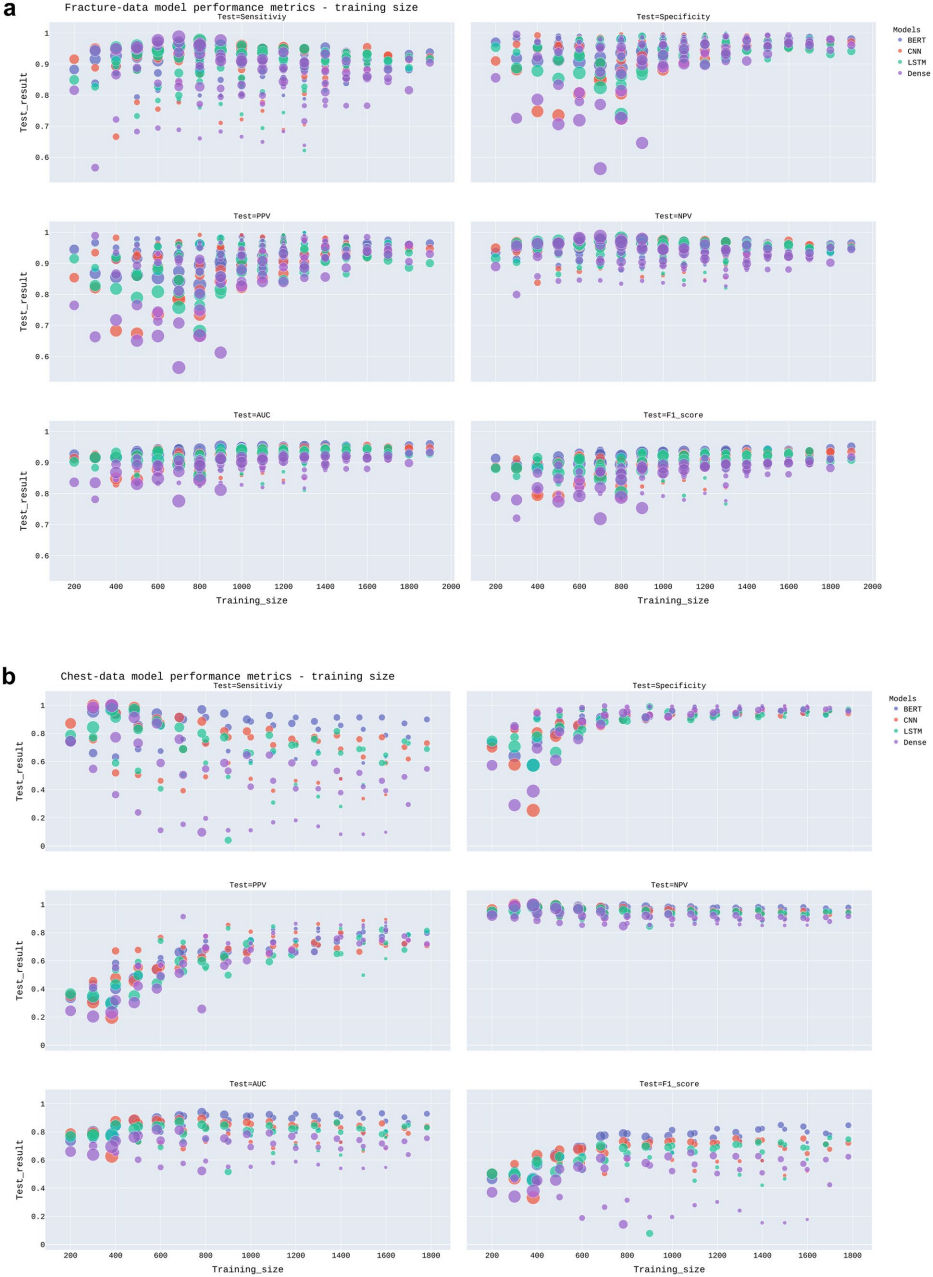
Scatterplot of model performance metrics (vertical axis) and training dataset size (horizontal axis) for (**a**) Fracture-data and (**b**) Chest-data. The size of the dots corresponds to the training dataset prevalence

**Fig. 4:**
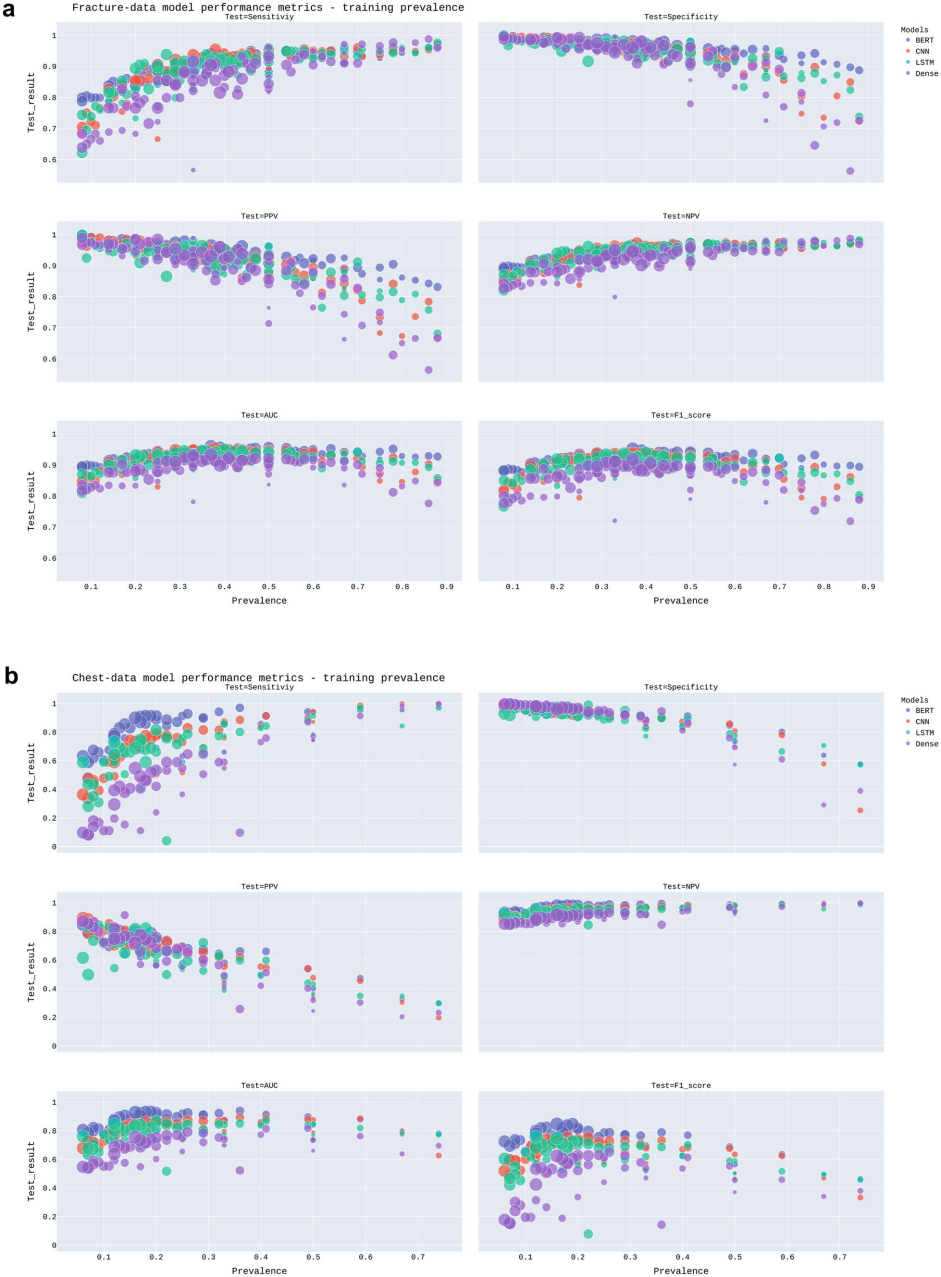
Scatterplot of model performance metrics (vertical axis) and prevalence (horizontal axis) for (**a**) Fracture-data and (**b**) Chest-data. The size of the dots corresponds to the training dataset size

Figures 3 and 4 demonstrate scatterplots for model performance metrics and training dataset size and prevalence, respectively. Model performance metrics on the test set ranged from 0.56–1.00 for the Fracture-data and from 0.04–1.00 for the Chest-data. Table 3 demonstrates the Pearson correlation coefficients between the performance metrics and training set size and prevalence, respectively. For both datasets, there is a strong negative correlation between prevalence and specificity and positive predictive value. The positive correlation between prevalence and sensitivity and the negative predictive value was strong in the Fracture-data set and moderate to strong in the Chest-data set. Size had only a strong positive correlation with specificity and PPV in the Chest-data set.

**Table 3:** Pearson correlation coefficients for training set size; prevalence and model performance metrics

**Fracture**	Size	Prevalence
Sensitivity	0,04	0,74
Specificity	0,36	-0,75
PPV	0,39	-0,80
NPV	0,05	0,74
AUC	0,36	0,16
F1_score	0,42	-0,02
**Chest**	Size	Prevalence
Sensitivity	-0,27	0,61
Specificity	0,60	-0,88
PPV	0,75	-0,88
NPV	-0,23	0,59
AUC	0,05	0,20
F1_score	0,28	-0,11

**Fig. 5:**
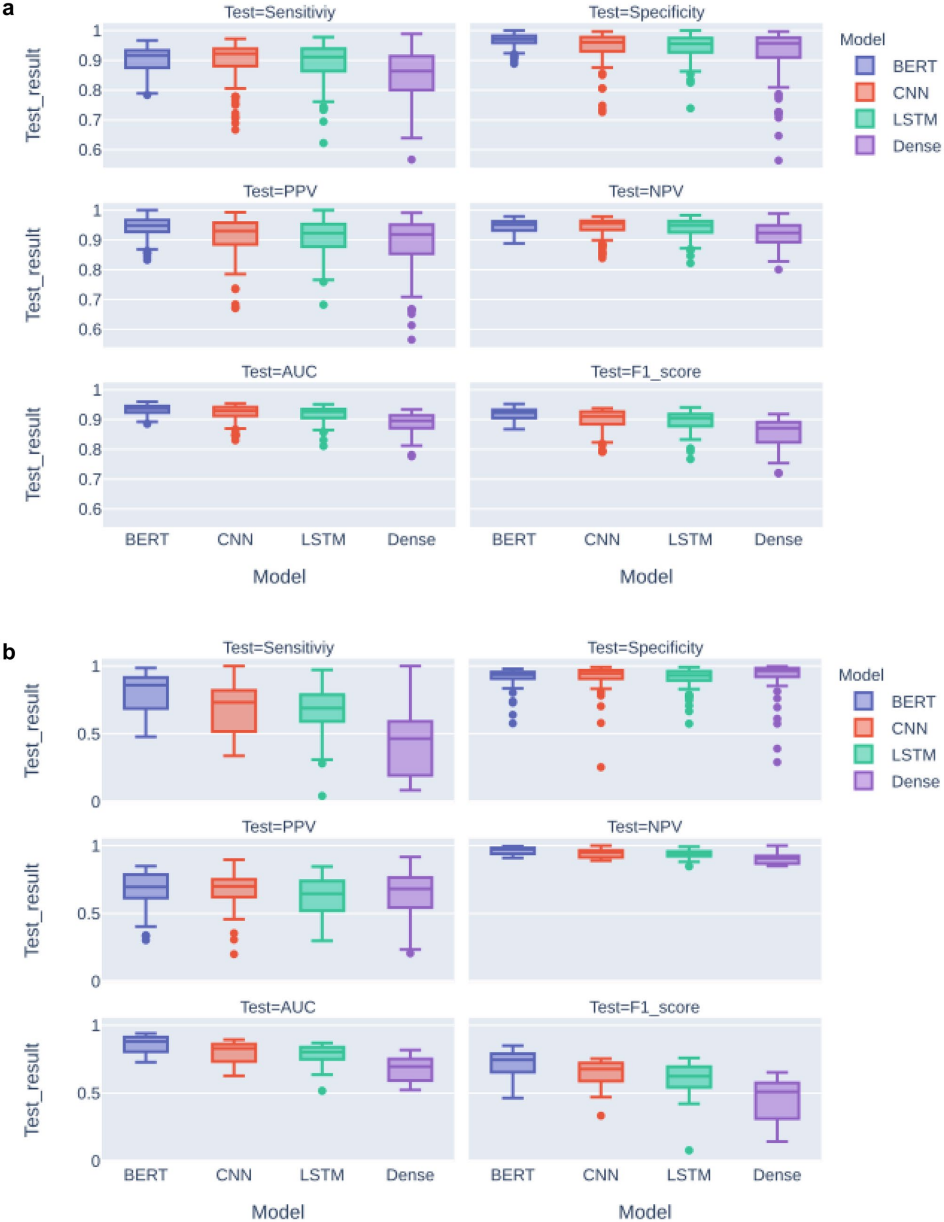
Boxplot of performance metrics per model for (**a**) Fracture-data and (**b**) Chest-data

In Fig. 5, performance metrics are summarized in boxplots.

In Table 4 (Fracture-data) and Table 5 (Chest-data), all pairs of models are compared for all performance metrics. For the Fracture-data, the BERT model outperforms the other models on most metrics, except the sensitivity and negative predicted value compared with LSTM and CNN. For the Chest-data, BERT outperforms the other models for sensitivity, npv, AUC, and F score. Specificity and ppv demonstrated no significant differences among the models. Table 6 highlights the most important findings.

**Table 4:** Comparison and t-test statistics of all performance metrics for all combinations of models trained on Fracture-data. The bold and underlined models have significantly better performance in the particular comparisons

**Metrics**	**Model 1**	**Model 2**	**tstat**	***p*-value**
Sensitivity	**BERT**	Dense	4.375	0.000
Sensitivity	BERT	LSTM	0.991	0.323
Sensitivity	BERT	CNN	0.553	0.581
Sensitivity	Dense	**LSTM**	-3.258	0.001
Sensitivity	Dense	**CNN**	-3.511	0.001
Sensitivity	LSTM	CNN	-0.358	0.721
Specificity	**BERT**	Dense	4.488	0.000
Specificity	**BERT**	LSTM	4.113	0.000
Specificity	**BERT**	CNN	3.447	0.001
Specificity	Dense	**LSTM**	-1.978	0.050
Specificity	Dense	CNN	-1.828	0.069
Specificity	LSTM	CNN	0.048	0.962
PPV	**BERT**	Dense	5.155	0.000
PPV	**BERT**	LSTM	4.465	0.000
PPV	**BERT**	CNN	3.552	0.000
PPV	Dense	LSTM	-1.936	0.055
PPV	Dense	**CNN**	-2.022	0.045
PPV	LSTM	CNN	-0.258	0.797
NPV	**BERT**	Dense	4.795	0.000
NPV	BERT	LSTM	1.138	0.257
NPV	BERT	CNN	0.620	0.536
NPV	Dense	**LSTM**	-3.513	0.001
NPV	Dense	**CNN**	-3.846	0.000
NPV	LSTM	CNN	-0.435	0.664
AUC	**BERT**	Dense	10.730	0.000
AUC	**BERT**	LSTM	4.541	0.000
AUC	**BERT**	CNN	3.618	0.000
AUC	Dense	**LSTM**	-6.329	0.000
AUC	Dense	**CNN**	-6.294	0.000
AUC	LSTM	CNN	-0.385	0.701
F1_score	**BERT**	Dense	11.362	0.000
F1_score	**BERT**	LSTM	5.608	0.000
F1_score	**BERT**	CNN	4.387	0.000
F1_score	Dense	**LSTM**	-6.205	0.000
F1_score	Dense	**CNN**	-6.171	0.000
F1_score	LSTM	CNN	-0.427	0.670

**Table 5:** Comparison and t-test statistics of all performance metrics for all combinations of models trained on Chest-data. The bold and underlined models have significantly better performance in the particular comparisons

**Metrics**	**Model 1**	**Model 2**	**t-stat**	***p*-value**
Sensitivity	**BERT**	CNN	3.559	0.001
Sensitivity	**BERT**	LSTM	4.493	0.000
Sensitivity	**BERT**	Dense	8.416	0.000
Sensitivity	CNN	LSTM	0.901	0.370
Sensitivity	**CNN**	Dense	5.151	0.000
Sensitivity	**LSTM**	Dense	4.333	0.000
Specificity	BERT	CNN	0.054	0.957
Specificity	BERT	LSTM	0.174	0.862
Specificity	BERT	Dense	0.138	0.890
Specificity	CNN	LSTM	0.088	0.930
Specificity	CNN	Dense	0.082	0.935
Specificity	LSTM	Dense	0.015	0.988
PPV	BERT	CNN	0.051	0.959
PPV	BERT	LSTM	1.401	0.165
PPV	BERT	Dense	1.046	0.299
PPV	CNN	LSTM	1.329	0.187
PPV	CNN	Dense	0.990	0.325
PPV	LSTM	Dense	-0.156	0.876
NPV	**BERT**	CNN	3.516	0.001
NPV	**BERT**	LSTM	4.821	0.000
NPV	**BERT**	Dense	9.064	0.000
NPV	CNN	LSTM	1.135	0.259
NPV	**CNN**	Dense	5.536	0.000
NPV	**LSTM**	Dense	4.561	0.000
AUC	**BERT**	CNN	4.269	0.000
AUC	**BERT**	LSTM	5.580	0.000
AUC	**BERT**	Dense	11.571	0.000
AUC	CNN	LSTM	1.243	0.217
AUC	**CNN**	Dense	7.349	0.000
AUC	**LSTM**	Dense	6.202	0.000
F1_score	**BERT**	CNN	3.485	0.001
F1_score	**BERT**	LSTM	4.690	0.000
F1_score	**BERT**	Dense	9.497	0.000
F1_score	CNN	LSTM	1.692	0.094
F1_score	**CNN**	Dense	7.140	0.000
F1_score	**LSTM**	Dense	5.334	0.000

**Table 6:** Results summary of model performance

**Model**	**Highlights**
All	• All models perform better on the shorter radiology reports of the Fracture-data than the more complex reports of the Chest-data. • Negative predictive value depends less on model type, training dataset size and prevalence than the positive predictive value
Dense	• Baseline model Dense performs well on the Fracture-data but depends more on variation in training dataset size and prevalence
LSTM / CNN	• The LSTM and CNN models demonstrate equal performance
BERT	• The BERT model has stable results despite a variation in training dataset size and prevalence. • The BERT model outperforms all other models, especially for the more complex reports of the Chest-data

## Discussion

In this study, we systematically evaluated the impact of training dataset size and prevalence, model type, and data complexity on the performance of four deep learning NLP models applied to radiology reports. The semi-automated pipeline allowed us to construct training sets of different sizes and different levels of class imbalance. This setup was chosen to discover the lower limit of usable dataset size and prevalence, as well as the limit above which adding more data had no added value. The results demonstrated that report complexity has a major impact on performance, illustrated by the substantially lower performance using the more complex dataset. For both datasets, the impact of training size and training prevalence demonstrated an identical pattern. Specificity and positive predictive value increased until there were about 800–1000 training samples; the values plateaued after that. Sensitivity and negative predictive value did not benefit substantially from an increase in the amount of training data. Prevalence correlated with sensitivity and positive predictive value and negatively correlated with specificity and negative predictive value. This aligns with the theoretically expected direction of effect, as explained in Appendix A3: In the case of class imbalance, the model tends to predict the majority class, and the positive and negative predicted values decrease by a reduction in false positive and false negative predictions, respectively.

The BERT model was the most stable algorithm in this study and demonstrated a limited impact of variation in data complexity, prevalence, and dataset size. BERT outperformed the other models on most metrics. The drawback of using BERT was the substantially longer training time of 30–60 min compared with less than 1 min for the other three algorithm types.

The conventional fully connected neural network (Dense) demonstrated the worst performance. The major difference between it and BERT, CNN, and LSTM was that the relationship between individual words was not taken into account. It is therefore not surprising that the nuances that radiologists embed in their reports are better extracted by the more advanced algorithm types.

To our knowledge, this is the first systematic, multifactorial comparative analysis of deep learning NLP in the field of radiology reporting. While no other study includes all the factors we investigated in ours, several authors describe one or more factors in their studies on natural language processing.

### Comparison of CNN and traditional NLP

Weikert et al. [35] compared two conventional NLP methods and a deep learning NLP (CNN) model and analyzed the impact of the amount of training data. The CNN outperformed the other models. Even though the authors also investigated chest radiology reports, the main difference was that they used only the impression section of the CT pulmonary angiography, which resulted in a more focused classification task. This could also explain the difference in the number of training reports needed to reach a plateau in performance (500 in their study compared to 800–1000 in our study).

Krsnik et al. [36] compared several traditional NLP methods with CNN when classifying knee MRI reports. The CNN outperformed the other techniques and had high performance (F1 score was 0.89–0.96) for the most represented conditions with a prevalence of 0.57–0.63. Conditions with lower prevalence were better detected with the conventional NLP methods. This illustrates the relationship between NLP model performance and prevalence and model type. Contrary to our study, no variation in prevalence was used.

Barash et al. [24] compared five different NLP algorithms, including four LSTM deep learning-based methods, and applied them to classify Hebrew language radiology reports in a general task (normal vs. abnormal, prevalence 46%) and a specific task (hemorrhage present or absent, prevalence 7%). The results were in the same range as our study, including lower sensitivity (66%–79%) and ppv (70%) and higher specificity and npv in the low-prevalence task, compared with the equal sensitivity/specificity (88%) and ppv/npv (90%) in the high-prevalence task.

### Comparison of LSTM and BERT

Datta et al. [37] applied several deep learning NLP methods to chest radiology reports where BERT outperformed LSTM. Instead of annotations at the report level (as in our study), they used annotations at the sentence level with words or a combination of words, not only to indicate diagnoses but also the spatial relation between any finding and its associated location. Because of this difference, the results are not directly comparable. The authors used under-sampling to deal with the substantial class imbalance at the sentence level in their data. In under-sampling, cases from the over-represented class are ignored in the training dataset. In fact, this is a variation of our approach with variation in the fraction of positive and negative cases to optimize model performance. At the level within the sentences, the authors described a higher performance for the words (5–6 times more frequent) with spatial information (F score 91.9–96.4) compared with less frequent words describing diagnoses (F score 75.2–82.8). Our study supports these results with a greater imbalance ratio and a greater performance difference.

### Comparison of different BERT models

Bressem et al. [38] compared four different BERT models, including RAD-BERT, that were specifically pre-trained on a large corpus of radiology texts. One of their other models was a pre-trained native language (German) model, just as we used a pre-trained Dutch model. Their analysis on the impact of variation in training set size for fine-tuning on model performance demonstrate a curve with a steep increase between 200–1000 cases, a gradual increase between 1000–2000, and a plateau in the 3000–4000 range. This is confirmed in our study. The different investigated items in the radiology reports had differences in prevalence and also different model performance metrics. This suggests a relation between performance and prevalence, but the authors did not vary the prevalence within the dataset, as we did in our study. The best-performing model demonstrated a best pooled auc of 0.98, compared with a best auc of 0.94 (Chest-data) and 0.96 (Fracture-data) in our study for the BERT model.

Our study and the referenced literature demonstrate the surprisingly high performance of deep learning NLP in radiology reporting. Information from both simple and more complex unstructured radiology reports can be extracted and used for downstream tasks such as epidemiological research, identification of incidental findings, assessment of diagnostic yield and imaging appropriateness, and labeling of images for training of computer vision algorithms [39–42].

### Limitations

The absence of inter-rater agreement assessment of the ground-truth annotations is a limitation. However, an unblinded assessment of the consistency of the annotations of the the Fracture-dataset by two radiologists and a blinded intra-rater agreement assessment of both datasets demonstrated excellent results.

Even though we constructed training sets with considerable variation in size and prevalence, the possible combinations were dependent on the original datasets' characteristics. The impact of variation in size and prevalence beyond these limits should be explored in further research.

Another limitation is that we investigated two report complexity levels but did not consider variation in report size within the datasets. Further research should elucidate to what extend NLP model performance depends on the size of radiology reports both in the training sets and the test sets. This is relevant because for clinical texts considerable larger than our current dataset research demonstrated a reduced performance of BERT compared with simpler architectures [43].

The results of our study are not directly generalizable to radiology reports from other institutions or other languages. External validation of the models should be performed to assess whether the results are generalizable to radiology reports from other institutions. Because BERT models are pre-trained on large datasets, and because our BERT model proved to deliver more stable results than the other models in our study, we expect a superior performance of BERT in the case of external validation.

## Conclusion

For NLP of radiology reports, all four model-architectures demonstrated high performance.

CNN, LSTM, and Dense were outperformed by the BERT algorithm because of its stable results, despite variation in training size and prevalence.

Awareness for variation in prevalence is warranted because this impacts sensitivity and specificity in opposite directions.

### Electronic Supplementary Material

Below is the link to the electronic supplementary material.

Supplementary file1 (PDF 146 KB)

Supplementary file2 (XLSX 306 KB)

Supplementary file3 (XLSX 4116 KB)

## Data Availability

Data available in supplementary material.

## Appendices

### A1 Annotation examples

- a. Fracture-dataset

Annotation purpose: identify reports with fractures or other traumatic abnormalities that require referral to the emergency department.

**Table:**

Positive example	Negative example
• Fracture	• No fracture
• Suspicion for a fracture	• Fracture unlikely
• Possible fracture	• No traumatic abnormalities
• Epiphysiolysis	• Normal findings
• Positive fat pad sign elbow	
• Luxation	

- b. Chest-dataset

Annotation purpose: identify reports with pulmonary infiltrates

**Table:**

Positive examples	Negative examples
Consolidation	No infiltrate
Infiltrate	No suspicion of an infiltrate
Possible infiltrate	Infiltrate unlikely
Maybe a small infiltrate	Normal findings
Suspicion of infiltrative abnormalitie	

### A2 Model architecture

Model: "Dense"

**Table:**

Layer (type)	Output Shape	Param #
Embedding (Embedding)	(None, 250, 32)	80000
flatten (Flatten)	(None, 8000)	0
Dense1 (Dense)	(None, 32)	256032
Dense-2 (Dense)	(None, 16)	528
Dense-3 (Dense)	(None, 8)	136
Dense-4 (Dense)	(None, 1)	9
Total params: 336,705		
Trainable params: 336,705		
Non-trainable params: 0		

Model: "LSTM"

**Table:**

Layer (type)	Output Shape	Param #
Embedding (Embedding)	(None, 250, 32)	80000
LSTM-1 (Bidirectional)	(None, 250, 64)	16640
LSTM-2 (Bidirectional)	(None, 64)	24832
Dense-1 (Dense)	(None, 24)	1560
Dense-2 (Dense)	(None, 1)	25
Total params: 123,057		
Trainable params: 123,057		
Non-trainable params: 0		

Model: "CNN"

**Table:**

Layer (type)	Output Shape	Param #
Embedding (Embedding)	(None, 250, 32)	80000
Conv-1D-1 (Conv1D)	(None, 246, 64)	10304
Pooling-1 (AveragePooling1D)	(None, 123, 64)	0
Conv-1D-2 (Conv1D)	(None, 119, 64)	20544
Pooling-2 (GlobalAveragePool)	(None, 64)	0
Dense-1 (Dense)	(None, 24)	1560
Dense-2 (Dense)	(None, 1)	25
Total params: 112,433		
Trainable params: 112,433		
Non-trainable params: 0		

### A3: Simulation of model training with neutral, positive, and negative class imbalance

True positive (TP), false negative (FN), false positive (FP), and true negative (TN) are presented in three scenarios to illustrate the impact of a change in sensitivity and specificity on the positive and negative predicted values. Compared to the baseline model (Scenario 1), the TP/FN/FP/TN values are manually changed to create an increase in sensitivity (Scenario 2) or an increase in specificity (Scenario 3) when applied to a test set with constant prevalence.

- PPV = positive predictive value = TP / ( TP + FP)
- NPV = negative predictive value = TN / ( TN + FN)
- sens = sensitivity = TP / ( TP + FN)
- spec = specificity = TN / ( TN + FP)

### Scenario 1: Normal training prevalence with an equal number of positive and negative cases. The result is equal sensitivity and specificity

**Table:**

	True							
Predicted	** + **		**-**				PPV	0.50
** + **	TP	80	FP	80	160		NPV	0.94
**-**	FN	20	TN	320	340		sens	0.80
		100		400			spec	0.80

### Scenario 2: With a high training prevalence, the model tends to predict a positive majority class. The result is high sensitivity and low specificity

**Table:**

	True							
Predicted	** + **		**-**				PPV	0.41
** + **	TP	99	FP	140	239		NPV	1.00
**-**	FN	1	TN	260	261		sens	0.99
		100		400			spec	0.65

### Scenario 3: With low training prevalence, the model tends to predict a negative majority class. The result is low sensitivity and high specificity

**Table:**

	True							
Predicted	** + **		**-**				PPV	0.98
** + **	TP	65	FP	1	66		NPV	0.92
**-**	FN	35	TN	399	434		sens	0.65
		100		400			spec	1.00

## Code availability

Code available in supplementary material as pdf.
