# Supplementary material for: Deep Learning-Based Natural Language Processing in Radiology: The Impact of Report Complexity, Disease Prevalence, Dataset Size, and Algorithm Type on Model Performance
Source: J Med Syst. 2021 Sep 4;45(10):91. doi: 10.1007/s10916-021-01761-4 (PMC8416876; doi:10.1007/s10916-021-01761-4)
Supplement: Supplementary file 1 — Supplementary file1 (PDF 146 KB) [file 10916_2021_1761_MOESM1_ESM.pdf]

NLP Radiology

Import Modules

In [1]:
```
!pip install simpletransformers
```

```
Requirement already satisfied: simpletransformers in c:\users\awolt\anaconda3\envs\nlp\lib\site-packages (0.2
9.1)
Requirement already satisfied: scikit-learn in c:\users\awolt\anaconda3\envs\nlp\lib\site-packages (from simp
letransformers) (0.23.1)
Requirement already satisfied: regex in c:\users\awolt\anaconda3\envs\nlp\lib\site-packages (from simpletrans
formers) (2020.5.14)
Requirement already satisfied: transformers>=2.9.1 in c:\users\awolt\anaconda3\envs\nlp\lib\site-packages (fr
om simpletransformers) (2.10.0)
Requirement already satisfied: seqeval in c:\users\awolt\anaconda3\envs\nlp\lib\site-packages (from simpletra
nsformers) (0.0.12)
Requirement already satisfied: tokenizers in c:\users\awolt\anaconda3\envs\nlp\lib\site-packages (from simple
transformers) (0.7.0)
Requirement already satisfied: tqdm in c:\users\awolt\anaconda3\envs\nlp\lib\site-packages (from simpletransf
ormers) (4.46.0)
Requirement already satisfied: scipy in c:\users\awolt\anaconda3\envs\nlp\lib\site-packages (from simpletrans
formers) (1.5.0)
Requirement already satisfied: requests in c:\users\awolt\anaconda3\envs\nlp\lib\site-packages (from simpletr
ansformers) (2.24.0)
Requirement already satisfied: pandas in c:\users\awolt\anaconda3\envs\nlp\lib\site-packages (from simpletran
sformers) (1.0.3)
Requirement already satisfied: numpy in c:\users\awolt\anaconda3\envs\nlp\lib\site-packages (from simpletrans
formers) (1.18.5)
Requirement already satisfied: tensorboardx in c:\users\awolt\anaconda3\envs\nlp\lib\site-packages (from simp
letransformers) (2.0)
Requirement already satisfied: joblib>=0.11 in c:\users\awolt\anaconda3\envs\nlp\lib\site-packages (from scik
it-learn->simpletransformers) (0.15.1)
Requirement already satisfied: threadpoolctl>=2.0.0 in c:\users\awolt\anaconda3\envs\nlp\lib\site-packages (f
rom scikit-learn->simpletransformers) (2.0.0)
Requirement already satisfied: sacremoses in c:\users\awolt\anaconda3\envs\nlp\lib\site-packages (from transf
ormers>=2.9.1->simpletransformers) (0.0.43)
Requirement already satisfied: filelock in c:\users\awolt\anaconda3\envs\nlp\lib\site-packages (from transfor
mers>=2.9.1->simpletransformers) (3.0.12)
Requirement already satisfied: sentencepiece in c:\users\awolt\anaconda3\envs\nlp\lib\site-packages (from tra
nsformers>=2.9.1->simpletransformers) (0.1.91)
Requirement already satisfied: Keras>=2.2.4 in c:\users\awolt\anaconda3\envs\nlp\lib\site-packages (from seqe
val->simpletransformers) (2.3.1)
Requirement already satisfied: certifi>=2017.4.17 in c:\users\awolt\anaconda3\envs\nlp\lib\site-packages (fro
m requests->simpletransformers) (2020.6.20)
Requirement already satisfied: chardet<4,>=3.0.2 in c:\users\awolt\anaconda3\envs\nlp\lib\site-packages (from
requests->simpletransformers) (3.0.4)
Requirement already satisfied: urllib3!=1.25.0,!=1.25.1,<1.26,>=1.21.1 in c:\users\awolt\anaconda3\envs\nlp\l
ib\site-packages (from requests->simpletransformers) (1.25.9)
Requirement already satisfied: idna<3,>=2.5 in c:\users\awolt\anaconda3\envs\nlp\lib\site-packages (from requ
ests->simpletransformers) (2.10)
Requirement already satisfied: python-dateutil>=2.6.1 in c:\users\awolt\anaconda3\envs\nlp\lib\site-packages
(from pandas->simpletransformers) (2.8.1)
Requirement already satisfied: pytz>=2017.2 in c:\users\awolt\anaconda3\envs\nlp\lib\site-packages (from pand
as->simpletransformers) (2020.1)
Requirement already satisfied: protobuf>=3.8.0 in c:\users\awolt\anaconda3\envs\nlp\lib\site-packages (from t
ensorboardx->simpletransformers) (3.12.3)
Requirement already satisfied: six in c:\users\awolt\anaconda3\envs\nlp\lib\site-packages (from tensorboardx-
>simpletransformers) (1.15.0)
Requirement already satisfied: click in c:\users\awolt\anaconda3\envs\nlp\lib\site-packages (from sacremoses-
>transformers>=2.9.1->simpletransformers) (7.1.2)
Requirement already satisfied: keras-preprocessing>=1.0.5 in c:\users\awolt\anaconda3\envs\nlp\lib\site-packa
ges (from Keras>=2.2.4->seqeval->simpletransformers) (1.1.0)
Requirement already satisfied: h5py in c:\users\awolt\anaconda3\envs\nlp\lib\site-packages (from Keras>=2.2.4
->seqeval->simpletransformers) (2.10.0)
Requirement already satisfied: keras-applications>=1.0.6 in c:\users\awolt\anaconda3\envs\nlp\lib\site-packag
es (from Keras>=2.2.4->seqeval->simpletransformers) (1.0.8)
Requirement already satisfied: pyyaml in c:\users\awolt\anaconda3\envs\nlp\lib\site-packages (from Keras>=2.
2.4->seqeval->simpletransformers) (5.3.1)
Requirement already satisfied: setuptools in c:\users\awolt\anaconda3\envs\nlp\lib\site-packages (from protob
uf>=3.8.0->tensorboardx->simpletransformers) (47.3.1.post20200622)
```

In [1]:
```
import numpy as np
import pandas as pd
import tensorflow as tf
from sklearn.utils import shuffle

from tensorflow.keras.preprocessing.text import Tokenizer
from tensorflow.keras.preprocessing.sequence import pad_sequences

from simpletransformers.classification import ClassificationModel

import logging

from datetime import datetime

from sklearn.metrics import balanced_accuracy_score
from sklearn.metrics import roc_auc_score
from sklearn.metrics import precision_recall_fscore_support
from sklearn.metrics import classification_report

import keras.backend as K
```

```
Using TensorFlow backend.
```

In [5]:
```
pip list
```

```
Package                Version
---------------------- -------------------
absl-py                0.9.0
astor                  0.8.0
async-generator        1.10
attrs                  19.3.0
backcall               0.2.0
bleach                 3.1.5
blinker                1.4Note: you may need to restart the kernel to use updated packages.

blis                   0.4.1
Brotli                 1.0.7
brotlipy               0.7.0
cachetools             4.1.0
catalogue              1.0.0
certifi                2020.6.20
cffi                   1.14.0
chardet                3.0.4
click                  7.1.2
colorama               0.4.3
cryptography           2.9.2
cymem                  2.0.3
dash                   1.13.4
dash-core-components   1.10.1
dash-html-components   1.0.3
dash-renderer          1.5.1
dash-table             4.8.1
decorator              4.4.2
defusedxml             0.6.0
entrypoints            0.3
et-xmlfile             1.0.1
filelock               3.0.12
Flask                  1.1.2
Flask-Compress         1.5.0
future                 0.18.2
gast                   0.2.2
google-auth            1.14.1
google-auth-oauthlib   0.4.1
google-pasta           0.2.0
grpcio                 1.27.2
h5py                   2.10.0
idna                   2.10
importlib-metadata     1.7.0
ipykernel              5.3.0
ipython                7.16.1
ipython-genutils       0.2.0
ipywidgets             7.5.1
itsdangerous           1.1.0
jdcal                  1.4.1
jedi                   0.17.1
Jinja2                 2.11.2
joblib                 0.15.1
jsonschema             3.2.0
jupyter                1.0.0
jupyter-client         6.1.3
jupyter-console        6.1.0
jupyter-core           4.6.3
jupyter-server         0.1.1
jupyterlab-pygments    0.1.1
Keras                  2.3.1
Keras-Applications     1.0.8
Keras-Preprocessing    1.1.0
Markdown               3.1.1
MarkupSafe             1.1.1
mistune                0.8.4
mkl-fft                1.1.0
mkl-random             1.1.1
mkl-service            2.3.0
murmurhash             1.0.2
nbconvert              5.6.1
nbformat               5.0.7
nl-core-news-sm        2.3.0
notebook               6.0.3
numpy                  1.18.5
oauthlib               3.1.0
openpyxl               3.0.3
opt-einsum             3.1.0
packaging              20.4
pandas                 1.0.3
pandocfilters          1.4.2
parso                  0.7.0
pickleshare            0.7.5
pip                    20.1.1
plac                   1.1.3
plotly                 4.8.1
preshed                3.0.2
prometheus-client      0.8.0
prompt-toolkit         3.0.5
protobuf               3.12.3
psutil                 5.7.0
pyasn1                 0.4.8
```

```
pyasn1-modules         0.2.7
pycparser              2.20
Pygments               2.6.1
PyJWT                  1.7.1
pyOpenSSL              19.1.0
pyparsing              2.4.7
pyreadline             2.1
pyrsistent             0.16.0
PySocks                1.7.1
python-dateutil        2.8.1
pytz                   2020.1
pywin32                227
pywinpty               0.5.7
PyYAML                 5.3.1
pyzmq                  19.0.1
qtconsole              4.7.5
QtPy                   1.9.0
regex                  2020.5.14
requests               2.24.0
requests-oauthlib      1.3.0
retrying               1.3.3
rsa                    4.0
sacremoses             0.0.43
scikit-learn           0.23.1
scikit-multilearn      0.2.0
scipy                  1.5.0
Send2Trash             1.5.0
sentencepiece          0.1.91
seqeval                0.0.12
setuptools             47.3.1.post20200622
simpletransformers     0.29.1
six                    1.15.0
spacy                  2.3.0
srsly                  1.0.2
tensorboard            2.2.1
tensorboard-plugin-wit 1.6.0
tensorboardX           2.0
tensorflow             2.1.0
tensorflow-estimator   2.1.0
termcolor              1.1.0
terminado              0.8.3
testpath               0.4.4
thinc                  7.4.1
threadpoolctl          2.0.0
tokenizers             0.7.0
torch                  1.3.1
tornado                6.0.4
tqdm                   4.46.0
traitlets              4.3.3
transformers           2.10.0
urllib3                1.25.9
voila                  0.1.21
voila-gridstack        0.0.9
wasabi                 0.7.0
wcwidth                0.2.5
webencodings           0.5.1
Werkzeug               1.0.1
wheel                  0.34.2
widgetsnbextension     3.5.1
win-inet-pton          1.1.0
wincertstore           0.2
wrapt                  1.12.1
xlrd                   1.2.0
zipp                   3.1.0
```

Preparation

Variables

In [2]:

```
# Define variables
vocab_size = 2500 #1000
embedding_dim = 32 #16
max_length = 250 #150 #120
trunc_type='post'
padding_type='post'
oov_tok = "<OOV>"
training_size_perc = 0.8 ##2200
num_epochs_number = 12
dataset_stepsize = 100 #250
dataset_stepsize_TEST = 40
use_small_sample_perc = 1 # < 1 to us small sample of dataset for testing purpose
path = '[path]'
```

Data

In [7]:
```
df = pd.read_excel(path+'/Data/Thorax2020_prevalproject.xlsx', 'Sheet1')
```

In [8]:
```
df = df[['ReportTextText', 'Result_Infiltraat']]
print(df)
```

```
                                         ReportTextText  Result_Infiltraat
0     X thorax 16-04-2020, 16:05\n\nHartgrootte is b...                  0
1     X thorax 16-04-2020, 11:14\n\nHartgrootte is b...                  0
2     X thorax 16-04-2020, 11:38\n\nLaagstaande, afg...                  0
3     CT thorax 16-04-2020, 21:41\n\nGeen pulmonale ...                  1
4     CT HR-thorax 16-04-2020, 13:07\nBlanco HRCT sc...                  1
...                                                 ...                ...
2250  CTA pulmonalis (longembolie) 14-04-2020, 16:21...                  0
2251  CTA pulmonalis (longembolie) 14-04-2020, 16:40...                  0
2252  CTA pulmonalis (longembolie) 15-04-2020, 06:10...                  0
2253  CTA pulmonalis (longembolie) 15-04-2020, 16:16...                  0
2254  CTA pulmonalis (longembolie) 15-04-2020, 19:43...                  0

[2255 rows x 2 columns]
```

In [13]:
```python
#prepare train-test-sets
#df pos neg split
df_pos = df.query('Result_Infiltraat == 1')
df_neg = df.query('Result_Infiltraat == 0')
# shuffle
df_pos_shuf = shuffle(df_pos)
df_neg_shuf = shuffle(df_neg)
#split train test
nr_pos = len(df_pos_shuf)
nr_neg = len(df_neg_shuf)
nr_train_pos = int(training_size_perc * nr_pos )
nr_train_neg = int(training_size_perc * nr_neg )
df_pos_TRAIN = df_pos_shuf.iloc[0:nr_train_pos]
df_pos_TEST =  df_pos_shuf.iloc[nr_train_pos:]
df_neg_TRAIN = df_neg_shuf.iloc[0:nr_train_neg]
df_neg_TEST = df_neg_shuf.iloc[nr_train_neg:]
df_TEST = pd.concat([df_pos_TEST, df_neg_TEST])

#safe dataset
Filename1 = 'df_TEST_THORAX_20201006'
df_TEST.to_excel(path+'/Jupyter_NLP_thoraxdataset/Data/'+Filename1+".xlsx")
Filename2 = 'df_pos_TRAIN_THORAX_20201006'
df_pos_TRAIN.to_excel(path+'/Jupyter_NLP_thoraxdataset/Data/'+Filename2+".xlsx")
Filename3= 'df_neg_TRAIN_THORAX_20201006'
df_neg_TRAIN.to_excel(path+'/Jupyter_NLP_thoraxdataset/Data/'+Filename3+".xlsx")


#def make_list_Pos_Neg_N(pos, neg, dataset_stepsize):
list_Pos_N = [*range(dataset_stepsize, nr_train_pos, dataset_stepsize)]
#list_Pos_N.append(pos) # add largest number of positive cases
list_Neg_N = [*range(dataset_stepsize, nr_train_neg, dataset_stepsize)]
#list_Neg_N.append(neg) # add largest number of negative cases
#return(list_Pos_N, list_Neg_N)


#prepare results dataframe
Training_combinations = pd.DataFrame(columns=['Dataset_ID', 'Pos', 'Neg', 'Training_size', 'Prevalence'])
teller=1
for i in list_Pos_N:
    for ii in list_Neg_N:
        ID = teller
        Pos = round(i ,0)
        Neg = round(ii, 0)
        Size = round((i + ii),0)
        Prev = round( (i/ (i + ii)), 2)
        Training_combinations.loc[teller] = (ID, Pos, Neg, Size, Prev)
        teller = teller + 1
print(Training_combinations)
Filename4 = 'Training_combinations_THORAX_20201006'
Training_combinations.to_excel(path+'/Jupyter_NLP_thoraxdataset/Data/'+Filename4+".xlsx")
#append info to results
```

```
    Dataset_ID    Pos     Neg  Training_size  Prevalence
1          1.0  100.0   100.0          200.0        0.50
2          2.0  100.0   200.0          300.0        0.33
3          3.0  100.0   300.0          400.0        0.25
4          4.0  100.0   400.0          500.0        0.20
5          5.0  100.0   500.0          600.0        0.17
6          6.0  100.0   600.0          700.0        0.14
7          7.0  100.0   700.0          800.0        0.12
8          8.0  100.0   800.0          900.0        0.11
9          9.0  100.0   900.0         1000.0        0.10
10        10.0  100.0  1000.0         1100.0        0.09
11        11.0  100.0  1100.0         1200.0        0.08
12        12.0  100.0  1200.0         1300.0        0.08
13        13.0  100.0  1300.0         1400.0        0.07
14        14.0  100.0  1400.0         1500.0        0.07
15        15.0  100.0  1500.0         1600.0        0.06
16        16.0  200.0   100.0          300.0        0.67
17        17.0  200.0   200.0          400.0        0.50
18        18.0  200.0   300.0          500.0        0.40
19        19.0  200.0   400.0          600.0        0.33
20        20.0  200.0   500.0          700.0        0.29
21        21.0  200.0   600.0          800.0        0.25
22        22.0  200.0   700.0          900.0        0.22
23        23.0  200.0   800.0         1000.0        0.20
24        24.0  200.0   900.0         1100.0        0.18
25        25.0  200.0  1000.0         1200.0        0.17
26        26.0  200.0  1100.0         1300.0        0.15
27        27.0  200.0  1200.0         1400.0        0.14
28        28.0  200.0  1300.0         1500.0        0.13
29        29.0  200.0  1400.0         1600.0        0.12
30        30.0  200.0  1500.0         1700.0        0.12
```

In [33]:
```
print(df)
```

```
                                           ReportTextText  Result_Infiltraat  \
0     X thorax 16-04-2020, 16:05\n\nHartgrootte is b...                  0
1     X thorax 16-04-2020, 11:14\n\nHartgrootte is b...                  0
2     X thorax 16-04-2020, 11:38\n\nLaagstaande, afg...                  0
3     CT thorax 16-04-2020, 21:41\n\nGeen pulmonale ...                  1
4     CT HR-thorax 16-04-2020, 13:07\nBlanco HRCT sc...                  1
...                                                 ...                ...
2250  CTA pulmonalis (longembolie) 14-04-2020, 16:21...                  0
2251  CTA pulmonalis (longembolie) 14-04-2020, 16:40...                  0
2252  CTA pulmonalis (longembolie) 15-04-2020, 06:10...                  0
2253  CTA pulmonalis (longembolie) 15-04-2020, 16:16...                  0
2254  CTA pulmonalis (longembolie) 15-04-2020, 19:43...                  0

      WordCount
0            26
1            28
2            44
3            66
4           250
...         ...
2250        108
2251         75
2252        136
2253         28
2254        214

[2255 rows x 3 columns]
```

In [9]:
```
#
df['WordCount'] = df['ReportTextText'].str.split().str.len()
```

In [28]:
```
df_WORDS = df['WordCount'].value_counts()
```

In [30]:
```
print(df_WORDS)
```

```
26     97
28     97
30     59
32     37
39     36
       ..
7       1
473     1
113     1
131     1
8       1
Name: WordCount, Length: 252, dtype: int64
```

In [23]:
```
import plotly.express as px
df.sort_values(by=['Result_Infiltraat'], inplace=True, ascending=False)
fig = px.histogram(df, x="WordCount", color="Result_Infiltraat")
fig.show()
```

In [7]:
```
print(df_TEST)
```

```
                                          ReportTextText  Result_Infiltraat
433   07-04-2020, 09:22, X thorax op zaal\n\nVergele...                  1
1365  X thorax 09-03-2020, 11:20\n\nIrregulaire cons...                  1
443   X thorax 07-04-2020, 11:02\n\nWordt vergeleken...                  1
620   Addendum: \nEchografisch onderzoek nadien toon...                  1
2233  CTA pulmonalis (longembolie) 09-04-2020, 19:35...                  1
...                                                 ...                ...
2113  03-02-2020, 11:56, X thorax\n\nVergelijk CT 20...                  0
1073  X thorax 11-03-2020, 15:34\n\nMatig ernstige h...                  0
344   CT thorax 08-04-2020, 17:38\n\nBlanco CT thora...                  0
930   X thorax 12-03-2020, 15:19\n\nHartgrootte is b...                  0
911   X thorax 12-03-2020, 08:24\n\nTer vergelijking...                  0

[452 rows x 2 columns]
```

In [8]:
```
print(list_Pos_N, list_Neg_N)
```

```
[100, 200] [100, 200, 300, 400, 500, 600, 700, 800, 900, 1000, 1100, 1200, 1300, 1400, 1500]
```

Models

In [3]:
```
def make_and_compile_models():
  model_dense = tf.keras.Sequential([
     tf.keras.layers.Embedding(vocab_size, embedding_dim, input_length=max_length, name='Embedding'),
     tf.keras.layers.Flatten(),
     tf.keras.layers.Dense(32, activation='relu', name='Dense1'),
     #tf.keras.layers.Dense(128, activation='relu'),
     #tf.keras.layers.Dropout(0.2),
     tf.keras.layers.Dense(16, activation='relu', name='Dense-2'),
     tf.keras.layers.Dense(8, activation='relu', name='Dense-3'), #24
     tf.keras.layers.Dense(1, activation='sigmoid', name='Dense-4')
  ])

  model_lstm = tf.keras.Sequential([
    tf.keras.layers.Embedding(vocab_size, embedding_dim, input_length=max_length, name='Embedding'),
    tf.keras.layers.Bidirectional(tf.keras.layers.LSTM(32, return_sequences=True), name='LSTM-1'),  #32
    tf.keras.layers.Bidirectional(tf.keras.layers.LSTM(32), name='LSTM-2'),
    tf.keras.layers.Dense(24, activation='relu', name='Dense-1'), #24
    tf.keras.layers.Dense(1, activation='sigmoid', name='Dense-2')
  ])

  model_cnn = tf.keras.Sequential([
    tf.keras.layers.Embedding(vocab_size, embedding_dim, input_length=max_length, name='Embedding'),
    tf.keras.layers.Conv1D(64, 5, activation='relu', name='Conv-1D-1'),  #32
    tf.keras.layers.AveragePooling1D(name='Pooling-1'),
    tf.keras.layers.Conv1D(64, 5, activation='relu', name='Conv-1D-2'),  #32
    #tf.keras.layers.AveragePooling1D(),
    #tf.keras.layers.Conv1D(32, 5, activation='relu'),  #32
    #tf.keras.layers.AveragePooling1D(),
    #tf.keras.layers.Conv1D(32, 5, activation='relu'),  #32
    tf.keras.layers.GlobalAveragePooling1D( name='Pooling-2'),
    tf.keras.layers.Dense(24, activation='relu',  name='Dense-1'), #24
    #tf.keras.layers.Dropout(0.2),
    #tf.keras.layers.Dense(12, activation='relu'),
    tf.keras.layers.Dense(1, activation='sigmoid',  name='Dense-2')
  ])


  model_dense.compile(loss='binary_crossentropy',optimizer='adam',metrics=['accuracy'])
  model_dense.summary()

  model_lstm.compile(loss='binary_crossentropy',optimizer='adam',metrics=['accuracy'])
  model_lstm.summary()

  model_cnn.compile(loss='binary_crossentropy',optimizer='adam',metrics=['accuracy'])
  model_cnn.summary()
  return(model_dense, model_lstm, model_cnn)
```

In [5]:
```
#BERT
def BERTmodel2(datastore_train, output_dir_bert):
  logging.basicConfig(level=logging.INFO)
  transformers_logger = logging.getLogger("transformers")
  transformers_logger.setLevel(logging.WARNING)
  # Create a ClassificationModel
  model_args = {
    "num_train_epochs": 4,
    "overwrite_output_dir": True,
    "save_model_every_epoch": False

}


  model_BERT = ClassificationModel('bert', 'wietsedv/bert-base-dutch-cased', args=model_args, use_cuda=False)
  # Train the model
  model_BERT.train_model(datastore_train, output_dir=output_dir_bert)  #other output_dir for every iteration
 in the loop
return( model_BERT)
```

Def's

In [6]:
```
def make_datastore_train(nr, Training_combinations, df_pos_TRAIN, df_neg_TRAIN):
    pos = Training_combinations.loc[nr]['Pos']
    neg = Training_combinations.loc[nr]['Neg']
    temp_pos = df_pos_TRAIN.loc[0:pos]
    temp_neg = df_neg_TRAIN.loc[0:neg]
    datastore_train = pd.concat([temp_pos, temp_neg])
    datastore_train = shuffle(datastore_train)
    return(datastore_train)

def make_trainset_from_datastore_train_and_testset_from_df_TEST(datastore_train, df_TEST):
  training_sentences_fixed = []
  training_labels_fixed = []
  #teller = 0
  for item in range(len(datastore_train)):
      #print(teller)
      #print('item=',item)
      temp_train = datastore_train.iloc[item]
      training_sentences_fixed.append(temp_train['ReportTextText'])
      #print('sentences=',sentences)
      training_labels_fixed.append(temp_train['Result_Infiltraat'])
      #print('labels=',labels)
      #teller = teller +1

  tokenizer = Tokenizer(num_words=vocab_size, oov_token=oov_tok)
  tokenizer.fit_on_texts(training_sentences_fixed)

  word_index = tokenizer.word_index

  training_sequences_fixed = tokenizer.texts_to_sequences(training_sentences_fixed)
  training_padded_fixed = pad_sequences(training_sequences_fixed, maxlen=max_length, padding=padding_type, tr
uncating=trunc_type)


#make test datasets with tokenized reports
  testing_sentences_fixed = []
  testing_labels_fixed = []
  for item in range(len(df_TEST)):
    temp_test = df_TEST.iloc[item]
    testing_sentences_fixed.append(temp_test['ReportTextText'])
    testing_labels_fixed.append(temp_test['Result_Infiltraat'])
     # tokenizer en word-index van trainingset
     #word_index = tokenizer.word_index van trainingset
    testing_sequences_fixed = tokenizer.texts_to_sequences(testing_sentences_fixed)
    testing_padded_fixed = pad_sequences(testing_sequences_fixed, maxlen=max_length, padding=padding_type, tr
uncating=trunc_type)
  Tokenizer_Ext = tokenizer
  return(training_padded_fixed, training_labels_fixed, testing_padded_fixed, testing_labels_fixed, Tokenizer_
Ext)

# train models(Dense, LSTM, CNN) and return histories
def train_models(training_padded, training_labels, testing_padded, testing_labels, model_dense, model_lstm, m
odel_cnn):
  num_epochs = num_epochs_number # 50
  training_padded = np.array(training_padded)
  training_labels = np.array(training_labels)
  testing_padded = np.array(testing_padded)
  testing_labels = np.array(testing_labels)
  history1 = model_dense.fit(training_padded, training_labels, epochs=num_epochs,  verbose=2, use_multiproces
sing = False)
  history2 = model_lstm.fit(training_padded, training_labels, epochs=num_epochs,  verbose=2, use_multiprocess
ing = False)
  history3 = model_cnn.fit(training_padded, training_labels, epochs=num_epochs,  verbose=2, use_multiprocessi
ng = False)
  return(history1, history2, history3)

#evaluation
def eval_model(model_nr, testing_padded_fixed, testing_labels_fixed):
  y_pred1 = model_nr.predict(testing_padded_fixed)
  y_true = testing_labels_fixed
  y_pred1_rounded = np.around(y_pred1)  #convert prediction to 0/1 labels
  precision, recall, fscore, support = precision_recall_fscore_support(y_true, y_pred1_rounded)
  fscore_0 = fscore[0]
  f1_score = fscore[1]
  npv = precision[0]
  ppv = precision[1]
  spec = recall[0]
  sens = recall[1]
  auc = roc_auc_score(y_true, y_pred1_rounded)
  return(sens, spec, ppv, npv, auc, f1_score)

#evaluate BERT
def evaluate_BERT(result_BERT, Count, DatasetN, PrevalenceN, Training_sizeN, Testing_sizeN ):
  tp = (result_bert['tp'])
  tn = (result_bert['tn'])
  fp = (result_bert['fp'])
  fn = (result_bert['fn'])
  #Evaluation_BERT = pd.DataFrame(columns=['ID','Dataset', 'Prevalence', 'Training_size', 'Testing_size', 'Mo
del', 'AUC', 'Recall_0', 'Recall_1', 'Precision_0', 'Precision_1', 'Fscore_0', 'Fscore_1', 'Balanced_accurac
y' ])
  Dataset = DatasetN
```

```
  balanced_accuracy_BERT = (1/2)*( (tp/(tp+fn)) + (tn/(tn+fp)))
  precision_BERT = tp / (tp+fp)
  recall_BERT = tp / (tp+fn)
  fscore_BERT = 2 * ( (precision_BERT * recall_BERT) / (precision_BERT + recall_BERT)  )
  # Evaluation_BERT is pd.dataframe that will be updated from this function (without input/export of this dat
aframe)
  Evaluation_BERT.loc[Count] = (Count, DatasetN, PrevalenceN, Training_sizeN, Testing_sizeN, 'BERT', 'auc', r
ecall_BERT, recall_BERT, precision_BERT, precision_BERT, fscore_BERT, fscore_BERT, balanced_accuracy_BERT)
  #let op: recall, precision en fscore niet apart voor 0 en 1.
  return() #dit was het
# return(sens, spec, ppv, npv, auc, f1_score) #dit moet het worden

#predict BERT (for evaluation)
def predictBERT(df_TEST, model):
    predictions, raw_outputs = model.predict(df_TEST)
    return(predictions, raw_outputs)


def evaluate_BERT2(y_true, y_pred):
    precision, recall, fscore, support = precision_recall_fscore_support(y_true, y_pred)
    fscore_0 = fscore[0]
    f1_score = fscore[1]
    npv = precision[0]
    ppv = precision[1]
    spec = recall[0]
    sens = recall[1]
    auc = roc_auc_score(y_true, y_pred)
    return(sens, spec, ppv, npv, auc, f1_score)
```

In [7]:
```
# overview of models
model_dense_graph, model_lstm_graph, model_cnn_graph = make_and_compile_models()
```

```
Model: "sequential"
_________________________________________________________________
Layer (type)                 Output Shape              Param #
=================================================================
Embedding (Embedding)        (None, 250, 32)           80000
_________________________________________________________________
flatten (Flatten)            (None, 8000)              0
_________________________________________________________________
Dense1 (Dense)               (None, 32)                256032
_________________________________________________________________
Dense-2 (Dense)              (None, 16)                528
_________________________________________________________________
Dense-3 (Dense)              (None, 8)                 136
_________________________________________________________________
Dense-4 (Dense)              (None, 1)                 9
=================================================================
Total params: 336,705
Trainable params: 336,705
Non-trainable params: 0
_________________________________________________________________
Model: "sequential_1"
_________________________________________________________________
Layer (type)                 Output Shape              Param #
=================================================================
Embedding (Embedding)        (None, 250, 32)           80000
_________________________________________________________________
LSTM-1 (Bidirectional)       (None, 250, 64)           16640
_________________________________________________________________
LSTM-2 (Bidirectional)       (None, 64)                24832
_________________________________________________________________
Dense-1 (Dense)              (None, 24)                1560
_________________________________________________________________
Dense-2 (Dense)              (None, 1)                 25
=================================================================
Total params: 123,057
Trainable params: 123,057
Non-trainable params: 0
_________________________________________________________________
Model: "sequential_2"
_________________________________________________________________
Layer (type)                 Output Shape              Param #
=================================================================
Embedding (Embedding)        (None, 250, 32)           80000
_________________________________________________________________
Conv-1D-1 (Conv1D)           (None, 246, 64)           10304
_________________________________________________________________
Pooling-1 (AveragePooling1D) (None, 123, 64)           0
_________________________________________________________________
Conv-1D-2 (Conv1D)           (None, 119, 64)           20544
_________________________________________________________________
Pooling-2 (GlobalAveragePool (None, 64)                0
_________________________________________________________________
Dense-1 (Dense)              (None, 24)                1560
_________________________________________________________________
Dense-2 (Dense)              (None, 1)                 25
=================================================================
Total params: 112,433
Trainable params: 112,433
Non-trainable params: 0
_________________________________________________________________
```

Experiments

In [34]:

```
#Dense, LSTM, CNN
Filename1 = 'df_TEST_THORAX_20201006'
Filename2 = 'df_pos_TRAIN_THORAX_20201006'
Filename3= 'df_neg_TRAIN_THORAX_20201006'
Filename4 = 'Training_combinations_THORAX_20201006'
Training_combinations = pd.read_excel(path+'/Jupyter_NLP_thoraxdataset/Data/'+Filename4+".xlsx")
df_TEST = pd.read_excel(path+'/Jupyter_NLP_thoraxdataset/Data/'+Filename1+".xlsx")
df_pos_TRAIN = pd.read_excel(path+'/Jupyter_NLP_thoraxdataset/Data/'+Filename2+".xlsx")
df_neg_TRAIN = pd.read_excel(path+'/Jupyter_NLP_thoraxdataset/Data/'+Filename3+".xlsx")
Evaluation = pd.DataFrame(columns=['ID','Nr', 'Training_size', 'Prevalence', 'Model', 'Sensitivity', 'Specifi
city', 'PPV', 'NPV', 'AUC', 'F1_score'])
eerste = 28  #
laatste = 29  # 1 extra dan einde


histories=pd.DataFrame()
Count = 0
for j in range(eerste, laatste):
    nr = j - 1

    datastore_train = make_datastore_train(nr, Training_combinations, df_pos_TRAIN, df_neg_TRAIN)
    training_padded_fixed, training_labels_fixed, testing_padded_fixed, testing_labels_fixed, Tokenizer_Ext =
make_trainset_from_datastore_train_and_testset_from_df_TEST(datastore_train, df_TEST)
    model_dense, model_lstm, model_cnn = make_and_compile_models()
    history1, history2, history3 = train_models(training_padded_fixed, training_labels_fixed, testing_padded_
fixed, testing_labels_fixed, model_dense, model_lstm, model_cnn)
    Models = [model_dense, model_lstm, model_cnn]
    Model_names = ['Dense', 'LSTM', 'CNN']
    prev = Training_combinations.loc[nr]['Prevalence']
    size = Training_combinations.loc[nr]['Training_size']
    print('prev=', prev)
    print('size=', size)
    print('nr=', nr)

    for iii in range(len(Models)):  #loop over model evaluation with prediction
        Count = Count+1
        print(Count)
        model = Models[iii]
        modelname = Model_names[iii]
        sens, spec, ppv, npv, auc, f1_score = eval_model(model, testing_padded_fixed, testing_labels_fixed)
        Evaluation.loc[Count] = (Count, j, size, prev, modelname, sens, spec, ppv, npv, auc, f1_score )
    now = datetime.now()
    dt_string = now.strftime("%Y%m%d_%H%M")
    filename5 = 'Evaluation_'+dt_string
    print('filename5=', filename5)
    Evaluation.to_excel(path+'/Jupyter_NLP_thoraxdataset/Data/History/'+filename5 +'.xlsx')
    hist1 = pd.DataFrame(history1.history)
    hist1['model']='Dense'
    hist1['size']=size
    hist1['prev']=prev
    hist2 = pd.DataFrame(history1.history)
    hist2['model']='LSTM'
    hist2['size']=size
    hist2['prev']=prev
    hist3 = pd.DataFrame(history1.history)
    hist3['model']='CNN'
    hist3['size']=size
    hist3['prev']=prev
    histories = pd.concat([histories, hist1, hist2, hist3])
    histories.to_excel(path+'/Jupyter_NLP_thoraxdataset/Data/History/'+'histories'+filename5+'.xlsx')
```

```
Model: "sequential_6"
_________________________________________________________________
Layer (type)                 Output Shape              Param #   
=================================================================
Embedding (Embedding)        (None, 250, 32)           80000     
_________________________________________________________________
flatten_2 (Flatten)          (None, 8000)              0         
_________________________________________________________________
Dense1 (Dense)               (None, 32)                256032    
_________________________________________________________________
Dense-2 (Dense)              (None, 16)                528       
_________________________________________________________________
Dense-3 (Dense)              (None, 8)                 136       
_________________________________________________________________
Dense-4 (Dense)              (None, 1)                 9         
=================================================================
Total params: 336,705
Trainable params: 336,705
Non-trainable params: 0
_________________________________________________________________
Model: "sequential_7"
_________________________________________________________________
Layer (type)                 Output Shape              Param #   
=================================================================
Embedding (Embedding)        (None, 250, 32)           80000     
_________________________________________________________________
LSTM-1 (Bidirectional)       (None, 250, 64)           16640     
_________________________________________________________________
LSTM-2 (Bidirectional)       (None, 64)                24832     
_________________________________________________________________
Dense-1 (Dense)              (None, 24)                1560      
_________________________________________________________________
Dense-2 (Dense)              (None, 1)                 25        
=================================================================
Total params: 123,057
Trainable params: 123,057
Non-trainable params: 0
_________________________________________________________________
Model: "sequential_8"
_________________________________________________________________
Layer (type)                 Output Shape              Param #   
=================================================================
Embedding (Embedding)        (None, 250, 32)           80000     
_________________________________________________________________
Conv-1D-1 (Conv1D)           (None, 246, 64)           10304     
_________________________________________________________________
Pooling-1 (AveragePooling1D) (None, 123, 64)           0         
_________________________________________________________________
Conv-1D-2 (Conv1D)           (None, 119, 64)           20544     
_________________________________________________________________
Pooling-2 (GlobalAveragePool (None, 64)                0         
_________________________________________________________________
Dense-1 (Dense)              (None, 24)                1560      
_________________________________________________________________
Dense-2 (Dense)              (None, 1)                 25        
=================================================================
Total params: 112,433
Trainable params: 112,433
Non-trainable params: 0
_________________________________________________________________
Train on 1502 samples
Epoch 1/12
1502/1502 - 1s - loss: 0.4543 - accuracy: 0.8375
Epoch 2/12
1502/1502 - 0s - loss: 0.3684 - accuracy: 0.8662
Epoch 3/12
1502/1502 - 0s - loss: 0.3252 - accuracy: 0.8675
Epoch 4/12
1502/1502 - 0s - loss: 0.2382 - accuracy: 0.8955
Epoch 5/12
1502/1502 - 0s - loss: 0.1114 - accuracy: 0.9607
Epoch 6/12
1502/1502 - 0s - loss: 0.0243 - accuracy: 0.9947
Epoch 7/12
1502/1502 - 1s - loss: 0.0046 - accuracy: 0.9993
Epoch 8/12
1502/1502 - 0s - loss: 0.0017 - accuracy: 1.0000
Epoch 9/12
1502/1502 - 0s - loss: 9.2999e-04 - accuracy: 1.0000
Epoch 10/12
1502/1502 - 0s - loss: 6.4206e-04 - accuracy: 1.0000
Epoch 11/12
1502/1502 - 0s - loss: 4.8488e-04 - accuracy: 1.0000
Epoch 12/12
1502/1502 - 0s - loss: 3.7545e-04 - accuracy: 1.0000
Train on 1502 samples
Epoch 1/12
1502/1502 - 8s - loss: 0.4720 - accuracy: 0.8655
Epoch 2/12
1502/1502 - 2s - loss: 0.3720 - accuracy: 0.8662
Epoch 3/12
```

```
1502/1502 - 2s - loss: 0.2335 - accuracy: 0.8955
Epoch 4/12
1502/1502 - 2s - loss: 0.1299 - accuracy: 0.9534
Epoch 5/12
1502/1502 - 2s - loss: 0.0768 - accuracy: 0.9727
Epoch 6/12
1502/1502 - 2s - loss: 0.0642 - accuracy: 0.9787
Epoch 7/12
1502/1502 - 2s - loss: 0.0839 - accuracy: 0.9720
Epoch 8/12
1502/1502 - 2s - loss: 0.0718 - accuracy: 0.9760
Epoch 9/12
1502/1502 - 2s - loss: 0.0541 - accuracy: 0.9814
Epoch 10/12
1502/1502 - 2s - loss: 0.0392 - accuracy: 0.9887
Epoch 11/12
1502/1502 - 2s - loss: 0.0216 - accuracy: 0.9947
Epoch 12/12
1502/1502 - 2s - loss: 0.0184 - accuracy: 0.9960
Train on 1502 samples
Epoch 1/12
1502/1502 - 2s - loss: 0.4393 - accuracy: 0.8662
Epoch 2/12
1502/1502 - 0s - loss: 0.3753 - accuracy: 0.8662
Epoch 3/12
1502/1502 - 0s - loss: 0.3436 - accuracy: 0.8662
Epoch 4/12
1502/1502 - 0s - loss: 0.2543 - accuracy: 0.8808
Epoch 5/12
1502/1502 - 0s - loss: 0.1396 - accuracy: 0.9454
Epoch 6/12
1502/1502 - 0s - loss: 0.0727 - accuracy: 0.9720
Epoch 7/12
1502/1502 - 0s - loss: 0.0377 - accuracy: 0.9880
Epoch 8/12
1502/1502 - 0s - loss: 0.0170 - accuracy: 0.9967
Epoch 9/12
1502/1502 - 0s - loss: 0.0092 - accuracy: 0.9973
Epoch 10/12
1502/1502 - 0s - loss: 0.0046 - accuracy: 0.9993
Epoch 11/12
1502/1502 - 0s - loss: 0.0028 - accuracy: 1.0000
Epoch 12/12
1502/1502 - 0s - loss: 0.0015 - accuracy: 1.0000
prev= 0.13
size= 1500.0
nr= 27
1
2
3
filename5= Evaluation_20201008_1527
```

In [31]:
```
#BERT
Filename1 = 'df_TEST_THORAX_20201006'
Filename2 = 'df_pos_TRAIN_THORAX_20201006'
Filename3= 'df_neg_TRAIN_THORAX_20201006'
Filename4 = 'Training_combinations_THORAX_20201006'
Training_combinations = pd.read_excel(path+'/Jupyter_NLP_thoraxdataset/Data/'+Filename4+".xlsx")
df_TEST = pd.read_excel(path+'/Jupyter_NLP_thoraxdataset/Data/'+Filename1+".xlsx")
df_pos_TRAIN = pd.read_excel(path+'/Jupyter_NLP_thoraxdataset/Data/'+Filename2+".xlsx")
df_neg_TRAIN = pd.read_excel(path+'/Jupyter_NLP_thoraxdataset/Data/'+Filename3+".xlsx")
Evaluation = pd.DataFrame(columns=['ID','Nr', 'Training_size', 'Prevalence', 'Model', 'Sensitivity', 'Specifi
city', 'PPV', 'NPV', 'AUC', 'F1_score'])
eerste = 1  #
laatste = 31  #
modelname = 'BERT'

Count = 0
for j in range(eerste, laatste):
    nr = j - 1
    Count = Count + 1
    datastore_train = make_datastore_train(nr, Training_combinations, df_pos_TRAIN, df_neg_TRAIN)
    datastore_train = datastore_train[['ReportTextText','Result_Infiltraat']]
    prev = Training_combinations.loc[nr]['Prevalence']
    size = Training_combinations.loc[nr]['Training_size']
    print('prev=', prev)
    print('size=', size)
    print('nr=', nr)
    output_dir_bert = "E:/NLP_models/BERT_prevalence_THORAX"
    df_TEST1 = df_TEST[['ReportTextText','Result_Infiltraat']]
    model_BERT = BERTmodel2(datastore_train, output_dir_bert)
    uitkomst, ruwe_data =    predictBERT(df_TEST['ReportTextText'], model_BERT)
    y_true = df_TEST['Result_Infiltraat']
    y_pred = pd.DataFrame(uitkomst)
    sens, spec, ppv, npv, auc, f1_score = evaluate_BERT2(y_true, y_pred)
    Evaluation.loc[Count] = (Count, j, size, prev, modelname, sens, spec, ppv, npv, auc, f1_score )
    now = datetime.now()
    dt_string = now.strftime("%Y%m%d_%H%M")
    filename5 = 'Evaluation_BERT'+dt_string
    print('filename5=', filename5)
    Evaluation.to_excel(path+'/Jupyter_NLP_thoraxdataset/Data/History/'+filename5 +'.xlsx')
```

```
prev= 0.5
size= 200.0
nr= 0

C:\Users\awolt\anaconda3\envs\NLP\lib\site-packages\simpletransformers\classification\classification_model.p
y:251: UserWarning:

Dataframe headers not specified. Falling back to using column 0 as text and column 1 as labels.

INFO:simpletransformers.classification.classification_model: Converting to features started. Cache is not use
d.



Running loss: 0.308128

Running loss: 0.175347

Running loss: 0.025715

Running loss: 0.023179

INFO:simpletransformers.classification.classification_model: Training of bert model complete. Saved to E:/NLP
_models/BERT_prevalence_THORAX.
INFO:simpletransformers.classification.classification_model: Converting to features started. Cache is not use
d.



filename5= Evaluation_BERT20201006_2022
prev= 0.33
size= 300.0
nr= 1

C:\Users\awolt\anaconda3\envs\NLP\lib\site-packages\simpletransformers\classification\classification_model.p
y:251: UserWarning:

Dataframe headers not specified. Falling back to using column 0 as text and column 1 as labels.

INFO:simpletransformers.classification.classification_model: Converting to features started. Cache is not use
d.



Running loss: 0.827608

Running loss: 0.850957

Running loss: 0.021440

Running loss: 0.013066

INFO:simpletransformers.classification.classification_model: Training of bert model complete. Saved to E:/NLP
_models/BERT_prevalence_THORAX.
INFO:simpletransformers.classification.classification_model: Converting to features started. Cache is not use
d.



filename5= Evaluation_BERT20201006_2041
prev= 0.25
size= 400.0
nr= 2

C:\Users\awolt\anaconda3\envs\NLP\lib\site-packages\simpletransformers\classification\classification_model.p
y:251: UserWarning:

Dataframe headers not specified. Falling back to using column 0 as text and column 1 as labels.

INFO:simpletransformers.classification.classification_model: Converting to features started. Cache is not use
d.



Running loss: 0.470993

Running loss: 0.037710

Running loss: 0.002272

Running loss: 0.003223
```

```
INFO:simpletransformers.classification.classification_model: Training of bert model complete. Saved to E:/NLP
_models/BERT_prevalence_THORAX.
INFO:simpletransformers.classification.classification_model: Converting to features started. Cache is not use
d.



filename5= Evaluation_BERT20201006_2102
prev= 0.2
size= 500.0
nr= 3

C:\Users\awolt\anaconda3\envs\NLP\lib\site-packages\simpletransformers\classification\classification_model.p
y:251: UserWarning:

Dataframe headers not specified. Falling back to using column 0 as text and column 1 as labels.

INFO:simpletransformers.classification.classification_model: Converting to features started. Cache is not use
d.




Running loss: 0.193534


Running loss: 0.051590


Running loss: 0.004970


Running loss: 0.004472


INFO:simpletransformers.classification.classification_model: Training of bert model complete. Saved to E:/NLP
_models/BERT_prevalence_THORAX.
INFO:simpletransformers.classification.classification_model: Converting to features started. Cache is not use
d.



filename5= Evaluation_BERT20201006_2131
prev= 0.17
size= 600.0
nr= 4

C:\Users\awolt\anaconda3\envs\NLP\lib\site-packages\simpletransformers\classification\classification_model.p
y:251: UserWarning:

Dataframe headers not specified. Falling back to using column 0 as text and column 1 as labels.

INFO:simpletransformers.classification.classification_model: Converting to features started. Cache is not use
d.




Running loss: 0.179947


Running loss: 2.804197


Running loss: 0.013142


Running loss: 0.003891


INFO:simpletransformers.classification.classification_model: Training of bert model complete. Saved to E:/NLP
_models/BERT_prevalence_THORAX.
INFO:simpletransformers.classification.classification_model: Converting to features started. Cache is not use
d.



filename5= Evaluation_BERT20201006_2201
prev= 0.14
size= 700.0
nr= 5

C:\Users\awolt\anaconda3\envs\NLP\lib\site-packages\simpletransformers\classification\classification_model.p
y:251: UserWarning:

Dataframe headers not specified. Falling back to using column 0 as text and column 1 as labels.

INFO:simpletransformers.classification.classification_model: Converting to features started. Cache is not use
d.




Running loss: 0.519240


Running loss: 0.443553
```

```
Running loss: 0.014828

Running loss: 0.001658

INFO:simpletransformers.classification.classification_model: Training of bert model complete. Saved to E:/NLP
_models/BERT_prevalence_THORAX.
INFO:simpletransformers.classification.classification_model: Converting to features started. Cache is not use
d.

filename5= Evaluation_BERT20201006_2238
prev= 0.12
size= 800.0
nr= 6

C:\Users\awolt\anaconda3\envs\NLP\lib\site-packages\simpletransformers\classification\classification_model.p
y:251: UserWarning:

Dataframe headers not specified. Falling back to using column 0 as text and column 1 as labels.

INFO:simpletransformers.classification.classification_model: Converting to features started. Cache is not use
d.

Running loss: 0.240730

Running loss: 1.746133

Running loss: 0.001955

Running loss: 0.000506

INFO:simpletransformers.classification.classification_model: Training of bert model complete. Saved to E:/NLP
_models/BERT_prevalence_THORAX.
INFO:simpletransformers.classification.classification_model: Converting to features started. Cache is not use
d.

filename5= Evaluation_BERT20201006_2329
prev= 0.11
size= 900.0
nr= 7

C:\Users\awolt\anaconda3\envs\NLP\lib\site-packages\simpletransformers\classification\classification_model.p
y:251: UserWarning:

Dataframe headers not specified. Falling back to using column 0 as text and column 1 as labels.

INFO:simpletransformers.classification.classification_model: Converting to features started. Cache is not use
d.

Running loss: 0.666798

Running loss: 0.050435

Running loss: 0.003770

Running loss: 0.001201

INFO:simpletransformers.classification.classification_model: Training of bert model complete. Saved to E:/NLP
_models/BERT_prevalence_THORAX.
INFO:simpletransformers.classification.classification_model: Converting to features started. Cache is not use
d.

filename5= Evaluation_BERT20201007_0014
prev= 0.1
size= 1000.0
nr= 8

C:\Users\awolt\anaconda3\envs\NLP\lib\site-packages\simpletransformers\classification\classification_model.p
y:251: UserWarning:

Dataframe headers not specified. Falling back to using column 0 as text and column 1 as labels.

INFO:simpletransformers.classification.classification_model: Converting to features started. Cache is not use
d.
```

```
Running loss: 0.023209

Running loss: 0.265384

Running loss: 0.000489

Running loss: 0.000250

INFO:simpletransformers.classification.classification_model: Training of bert model complete. Saved to E:/NLP
_models/BERT_prevalence_THORAX.
INFO:simpletransformers.classification.classification_model: Converting to features started. Cache is not use
d.

filename5= Evaluation_BERT20201007_0059
prev= 0.09
size= 1100.0
nr= 9

C:\Users\awolt\anaconda3\envs\NLP\lib\site-packages\simpletransformers\classification\classification_model.p
y:251: UserWarning:

Dataframe headers not specified. Falling back to using column 0 as text and column 1 as labels.

INFO:simpletransformers.classification.classification_model: Converting to features started. Cache is not use
d.

Running loss: 0.536797

Running loss: 0.286102

Running loss: 0.002303

Running loss: 0.003978

INFO:simpletransformers.classification.classification_model: Training of bert model complete. Saved to E:/NLP
_models/BERT_prevalence_THORAX.
INFO:simpletransformers.classification.classification_model: Converting to features started. Cache is not use
d.

filename5= Evaluation_BERT20201007_0145
prev= 0.08
size= 1200.0
nr= 10

C:\Users\awolt\anaconda3\envs\NLP\lib\site-packages\simpletransformers\classification\classification_model.p
y:251: UserWarning:

Dataframe headers not specified. Falling back to using column 0 as text and column 1 as labels.

INFO:simpletransformers.classification.classification_model: Converting to features started. Cache is not use
d.

Running loss: 1.652708

Running loss: 1.557388

Running loss: 0.000881

Running loss: 0.000416

INFO:simpletransformers.classification.classification_model: Training of bert model complete. Saved to E:/NLP
_models/BERT_prevalence_THORAX.
INFO:simpletransformers.classification.classification_model: Converting to features started. Cache is not use
d.

filename5= Evaluation_BERT20201007_0235
prev= 0.08
size= 1300.0
nr= 11
```

```
C:\Users\awolt\anaconda3\envs\NLP\lib\site-packages\simpletransformers\classification\classification_model.p
y:251: UserWarning:

Dataframe headers not specified. Falling back to using column 0 as text and column 1 as labels.

INFO:simpletransformers.classification.classification_model: Converting to features started. Cache is not use
d.

Running loss: 0.533506

Running loss: 0.019977

Running loss: 0.002350

Running loss: 0.001026

INFO:simpletransformers.classification.classification_model: Training of bert model complete. Saved to E:/NLP
_models/BERT_prevalence_THORAX.
INFO:simpletransformers.classification.classification_model: Converting to features started. Cache is not use
d.

filename5= Evaluation_BERT20201007_0329
prev= 0.07
size= 1400.0
nr= 12

C:\Users\awolt\anaconda3\envs\NLP\lib\site-packages\simpletransformers\classification\classification_model.p
y:251: UserWarning:

Dataframe headers not specified. Falling back to using column 0 as text and column 1 as labels.

INFO:simpletransformers.classification.classification_model: Converting to features started. Cache is not use
d.

Running loss: 0.031207

Running loss: 0.001036

Running loss: 0.001124

Running loss: 0.000311

INFO:simpletransformers.classification.classification_model: Training of bert model complete. Saved to E:/NLP
_models/BERT_prevalence_THORAX.
INFO:simpletransformers.classification.classification_model: Converting to features started. Cache is not use
d.

filename5= Evaluation_BERT20201007_0433
prev= 0.07
size= 1500.0
nr= 13

C:\Users\awolt\anaconda3\envs\NLP\lib\site-packages\simpletransformers\classification\classification_model.p
y:251: UserWarning:

Dataframe headers not specified. Falling back to using column 0 as text and column 1 as labels.

INFO:simpletransformers.classification.classification_model: Converting to features started. Cache is not use
d.

Running loss: 0.452895

Running loss: 0.002826

Running loss: 0.001601

Running loss: 0.000327

INFO:simpletransformers.classification.classification_model: Training of bert model complete. Saved to E:/NLP
_models/BERT_prevalence_THORAX.
INFO:simpletransformers.classification.classification_model: Converting to features started. Cache is not use
d.
```

```
filename5= Evaluation_BERT20201007_0536
prev= 0.06
size= 1600.0
nr= 14

C:\Users\awolt\anaconda3\envs\NLP\lib\site-packages\simpletransformers\classification\classification_model.p
y:251: UserWarning:

Dataframe headers not specified. Falling back to using column 0 as text and column 1 as labels.

INFO:simpletransformers.classification.classification_model: Converting to features started. Cache is not use
d.



Running loss: 0.026800


Running loss: 0.017476


Running loss: 0.000615


Running loss: 0.000336


INFO:simpletransformers.classification.classification_model: Training of bert model complete. Saved to E:/NLP
_models/BERT_prevalence_THORAX.
INFO:simpletransformers.classification.classification_model: Converting to features started. Cache is not use
d.



filename5= Evaluation_BERT20201007_0643
prev= 0.67
size= 300.0
nr= 15

C:\Users\awolt\anaconda3\envs\NLP\lib\site-packages\simpletransformers\classification\classification_model.p
y:251: UserWarning:

Dataframe headers not specified. Falling back to using column 0 as text and column 1 as labels.

INFO:simpletransformers.classification.classification_model: Converting to features started. Cache is not use
d.



Running loss: 0.619967


Running loss: 0.586118


Running loss: 0.189749


Running loss: 0.007092


INFO:simpletransformers.classification.classification_model: Training of bert model complete. Saved to E:/NLP
_models/BERT_prevalence_THORAX.
INFO:simpletransformers.classification.classification_model: Converting to features started. Cache is not use
d.



filename5= Evaluation_BERT20201007_0657
prev= 0.5
size= 400.0
nr= 16

C:\Users\awolt\anaconda3\envs\NLP\lib\site-packages\simpletransformers\classification\classification_model.p
y:251: UserWarning:

Dataframe headers not specified. Falling back to using column 0 as text and column 1 as labels.

INFO:simpletransformers.classification.classification_model: Converting to features started. Cache is not use
d.



Running loss: 0.325808


Running loss: 0.114415


Running loss: 0.140266


Running loss: 0.006106
```

```
INFO:simpletransformers.classification.classification_model: Training of bert model complete. Saved to E:/NLP
_models/BERT_prevalence_THORAX.
INFO:simpletransformers.classification.classification_model: Converting to features started. Cache is not use
d.
```

```
filename5= Evaluation_BERT20201007_0716
prev= 0.4
size= 500.0
nr= 17
```

```
C:\Users\awolt\anaconda3\envs\NLP\lib\site-packages\simpletransformers\classification\classification_model.p
y:251: UserWarning:

Dataframe headers not specified. Falling back to using column 0 as text and column 1 as labels.

INFO:simpletransformers.classification.classification_model: Converting to features started. Cache is not use
d.
```

```
Running loss: 0.600499

Running loss: 0.225614

Running loss: 0.009339

Running loss: 0.002663
```

```
INFO:simpletransformers.classification.classification_model: Training of bert model complete. Saved to E:/NLP
_models/BERT_prevalence_THORAX.
INFO:simpletransformers.classification.classification_model: Converting to features started. Cache is not use
d.
```

```
filename5= Evaluation_BERT20201007_0738
prev= 0.33
size= 600.0
nr= 18
```

```
C:\Users\awolt\anaconda3\envs\NLP\lib\site-packages\simpletransformers\classification\classification_model.p
y:251: UserWarning:

Dataframe headers not specified. Falling back to using column 0 as text and column 1 as labels.

INFO:simpletransformers.classification.classification_model: Converting to features started. Cache is not use
d.
```

```
Running loss: 0.757230

Running loss: 0.044053

Running loss: 2.303625

Running loss: 0.000515
```

```
INFO:simpletransformers.classification.classification_model: Training of bert model complete. Saved to E:/NLP
_models/BERT_prevalence_THORAX.
INFO:simpletransformers.classification.classification_model: Converting to features started. Cache is not use
d.
```

```
filename5= Evaluation_BERT20201007_0804
prev= 0.29
size= 700.0
nr= 19
```

```
C:\Users\awolt\anaconda3\envs\NLP\lib\site-packages\simpletransformers\classification\classification_model.p
y:251: UserWarning:

Dataframe headers not specified. Falling back to using column 0 as text and column 1 as labels.

INFO:simpletransformers.classification.classification_model: Converting to features started. Cache is not use
d.
```

```
Running loss: 0.748764

Running loss: 0.025750
```

```
Running loss: 0.216078

Running loss: 0.002271

INFO:simpletransformers.classification.classification_model: Training of bert model complete. Saved to E:/NLP
_models/BERT_prevalence_THORAX.
INFO:simpletransformers.classification.classification_model: Converting to features started. Cache is not use
d.

filename5= Evaluation_BERT20201007_0834
prev= 0.25
size= 800.0
nr= 20

C:\Users\awolt\anaconda3\envs\NLP\lib\site-packages\simpletransformers\classification\classification_model.p
y:251: UserWarning:

Dataframe headers not specified. Falling back to using column 0 as text and column 1 as labels.

INFO:simpletransformers.classification.classification_model: Converting to features started. Cache is not use
d.

Running loss: 0.984572

Running loss: 0.001768

Running loss: 0.000466

Running loss: 0.001745

INFO:simpletransformers.classification.classification_model: Training of bert model complete. Saved to E:/NLP
_models/BERT_prevalence_THORAX.
INFO:simpletransformers.classification.classification_model: Converting to features started. Cache is not use
d.

filename5= Evaluation_BERT20201007_0909
prev= 0.22
size= 900.0
nr= 21

C:\Users\awolt\anaconda3\envs\NLP\lib\site-packages\simpletransformers\classification\classification_model.p
y:251: UserWarning:

Dataframe headers not specified. Falling back to using column 0 as text and column 1 as labels.

INFO:simpletransformers.classification.classification_model: Converting to features started. Cache is not use
d.

Running loss: 0.529352

Running loss: 0.014645

Running loss: 0.001633

Running loss: 0.002405

INFO:simpletransformers.classification.classification_model: Training of bert model complete. Saved to E:/NLP
_models/BERT_prevalence_THORAX.
INFO:simpletransformers.classification.classification_model: Converting to features started. Cache is not use
d.

filename5= Evaluation_BERT20201007_0947
prev= 0.2
size= 1000.0
nr= 22

C:\Users\awolt\anaconda3\envs\NLP\lib\site-packages\simpletransformers\classification\classification_model.p
y:251: UserWarning:

Dataframe headers not specified. Falling back to using column 0 as text and column 1 as labels.

INFO:simpletransformers.classification.classification_model: Converting to features started. Cache is not use
d.
```

```
Running loss: 0.013972

Running loss: 0.001922

Running loss: 0.000601

Running loss: 0.001160

INFO:simpletransformers.classification.classification_model: Training of bert model complete. Saved to E:/NLP
_models/BERT_prevalence_THORAX.
INFO:simpletransformers.classification.classification_model: Converting to features started. Cache is not use
d.

filename5= Evaluation_BERT20201007_1030
prev= 0.18
size= 1100.0
nr= 23

C:\Users\awolt\anaconda3\envs\NLP\lib\site-packages\simpletransformers\classification\classification_model.p
y:251: UserWarning:

Dataframe headers not specified. Falling back to using column 0 as text and column 1 as labels.

INFO:simpletransformers.classification.classification_model: Converting to features started. Cache is not use
d.

Running loss: 0.066426

Running loss: 0.002142

Running loss: 0.000838

Running loss: 0.000313

INFO:simpletransformers.classification.classification_model: Training of bert model complete. Saved to E:/NLP
_models/BERT_prevalence_THORAX.
INFO:simpletransformers.classification.classification_model: Converting to features started. Cache is not use
d.

filename5= Evaluation_BERT20201007_1117
prev= 0.17
size= 1200.0
nr= 24

C:\Users\awolt\anaconda3\envs\NLP\lib\site-packages\simpletransformers\classification\classification_model.p
y:251: UserWarning:

Dataframe headers not specified. Falling back to using column 0 as text and column 1 as labels.

INFO:simpletransformers.classification.classification_model: Converting to features started. Cache is not use
d.

Running loss: 1.995735

Running loss: 0.035820

Running loss: 0.008260

Running loss: 0.000399

INFO:simpletransformers.classification.classification_model: Training of bert model complete. Saved to E:/NLP
_models/BERT_prevalence_THORAX.
INFO:simpletransformers.classification.classification_model: Converting to features started. Cache is not use
d.

filename5= Evaluation_BERT20201007_1913
prev= 0.15
size= 1300.0
nr= 25
```

```
C:\Users\awolt\anaconda3\envs\NLP\lib\site-packages\simpletransformers\classification\classification_model.p
y:251: UserWarning:

Dataframe headers not specified. Falling back to using column 0 as text and column 1 as labels.

INFO:simpletransformers.classification.classification_model: Converting to features started. Cache is not use
d.



Running loss: 0.332261

Running loss: 0.010098

Running loss: 0.001724

Running loss: 0.001555

INFO:simpletransformers.classification.classification_model: Training of bert model complete. Saved to E:/NLP
_models/BERT_prevalence_THORAX.
INFO:simpletransformers.classification.classification_model: Converting to features started. Cache is not use
d.


filename5= Evaluation_BERT20201007_2007
prev= 0.14
size= 1400.0
nr= 26

C:\Users\awolt\anaconda3\envs\NLP\lib\site-packages\simpletransformers\classification\classification_model.p
y:251: UserWarning:

Dataframe headers not specified. Falling back to using column 0 as text and column 1 as labels.

INFO:simpletransformers.classification.classification_model: Converting to features started. Cache is not use
d.



Running loss: 0.005378

Running loss: 0.023324

Running loss: 0.004519

Running loss: 0.016370

INFO:simpletransformers.classification.classification_model: Training of bert model complete. Saved to E:/NLP
_models/BERT_prevalence_THORAX.
INFO:simpletransformers.classification.classification_model: Converting to features started. Cache is not use
d.


filename5= Evaluation_BERT20201007_2105
prev= 0.13
size= 1500.0
nr= 27

C:\Users\awolt\anaconda3\envs\NLP\lib\site-packages\simpletransformers\classification\classification_model.p
y:251: UserWarning:

Dataframe headers not specified. Falling back to using column 0 as text and column 1 as labels.

INFO:simpletransformers.classification.classification_model: Converting to features started. Cache is not use
d.



Running loss: 0.113475

Running loss: 0.004722

Running loss: 0.002005

Running loss: 0.002766

INFO:simpletransformers.classification.classification_model: Training of bert model complete. Saved to E:/NLP
_models/BERT_prevalence_THORAX.
INFO:simpletransformers.classification.classification_model: Converting to features started. Cache is not use
d.
```

```
filename5= Evaluation_BERT20201007_2206
prev= 0.12
size= 1600.0
nr= 28

C:\Users\awolt\anaconda3\envs\NLP\lib\site-packages\simpletransformers\classification\classification_model.p
y:251: UserWarning:

Dataframe headers not specified. Falling back to using column 0 as text and column 1 as labels.

INFO:simpletransformers.classification.classification_model: Converting to features started. Cache is not use
d.



Running loss: 0.063189

Running loss: 0.012750

Running loss: 0.000245

Running loss: 0.000233


INFO:simpletransformers.classification.classification_model: Training of bert model complete. Saved to E:/NLP
_models/BERT_prevalence_THORAX.
INFO:simpletransformers.classification.classification_model: Converting to features started. Cache is not use
d.



filename5= Evaluation_BERT20201007_2313
prev= 0.12
size= 1700.0
nr= 29

C:\Users\awolt\anaconda3\envs\NLP\lib\site-packages\simpletransformers\classification\classification_model.p
y:251: UserWarning:

Dataframe headers not specified. Falling back to using column 0 as text and column 1 as labels.

INFO:simpletransformers.classification.classification_model: Converting to features started. Cache is not use
d.



Running loss: 0.003821

Running loss: 0.003158

Running loss: 0.000415

Running loss: 0.000761


INFO:simpletransformers.classification.classification_model: Training of bert model complete. Saved to E:/NLP
_models/BERT_prevalence_THORAX.
INFO:simpletransformers.classification.classification_model: Converting to features started. Cache is not use
d.



filename5= Evaluation_BERT20201008_0023
```
